# Supplementary material for: A robust cis-Mendelian randomization method with application to drug target discovery
Source: Nat Commun. 2024 Jul 18;15:6072. doi: 10.1038/s41467-024-50385-y (PMC11258283; doi:10.1038/s41467-024-50385-y)
Supplement: Supplementary file 1 — Supplementary Information [file 41467_2024_50385_MOESM1_ESM.pdf]

Supplementary materials: A robust cis-Mendelian randomization method with application to drug target discovery

S1 Proof of Theorem 1

Recall that our log-likelihood for the proposed model (up to some constant) is:

$$l(\theta, \mathbf{b}_X, \mathbf{r}; \hat{\boldsymbol{\beta}}_X, \hat{\boldsymbol{\beta}}_Y, \boldsymbol{\Sigma}_X, \boldsymbol{\Sigma}_Y) = -\frac{1}{2}\Big[(\hat{\boldsymbol{\beta}}_X - \mathbf{b}_X)^T \boldsymbol{\Sigma}_X^{-1}(\hat{\boldsymbol{\beta}}_X - \mathbf{b}_X) + (\hat{\boldsymbol{\beta}}_Y - \theta\mathbf{b}_X - \mathbf{r})^T \boldsymbol{\Sigma}_Y^{-1}(\hat{\boldsymbol{\beta}}_Y - \theta\mathbf{b}_X - \mathbf{r})\Big]. \quad (1)$$

We will first give the algorithm to solving $\{\hat{\theta}, \hat{\mathbf{b}}_X, \hat{\mathbf{r}}\} = \arg\max_{\theta, \mathbf{b}_X, \mathbf{r}} l(\theta, \mathbf{b}_X, \mathbf{r})$ under the constraint of $r_i = 0$ if $i \in \mathcal{I}$, where $\mathcal{I}$ is the set of unselected variables in Algorithm 1. We introduce the notation of $\mathbf{a}_{\mathcal{A}} = (a_i, i \in \mathcal{A}) \in \mathbb{R}^{|\mathcal{A}|}$ be a sub-vector of $\mathbf{a}$. For the simplicity of notation and without loss of generality, we assume $\mathcal{A} = \{1, 2, \ldots, K\}$, $\mathcal{I} = \{K+1, \ldots, m\}$. The first-order partial derivatives of l are given by:

$$\frac{\partial l}{\partial \mathbf{r}_{\mathcal{A}}} = (\hat{\boldsymbol{\beta}}_Y^T - \theta\mathbf{b}_X^T - \mathbf{r}^T)\boldsymbol{\Sigma}_Y^{-1}\begin{bmatrix}\mathbf{I}_K \\ \mathbf{0}_{(m-K)\times K}\end{bmatrix},$$
$$\frac{\partial l}{\partial \mathbf{b}_X} = \theta(\hat{\boldsymbol{\beta}}_Y^T - \theta\mathbf{b}_X^T - \mathbf{r}^T)\boldsymbol{\Sigma}_Y^{-1} + (\hat{\boldsymbol{\beta}}_X^T - \mathbf{b}_X^T)\boldsymbol{\Sigma}_X^{-1},$$
$$\frac{\partial l}{\partial \theta} = (\hat{\boldsymbol{\beta}}_Y^T - \theta\mathbf{b}_X^T - \mathbf{r}^T)\boldsymbol{\Sigma}_Y^{-1}\mathbf{b}_X,$$

Equate the above partial derivatives of log-likelihood to 0, we have

$$\mathbf{r}_{\mathcal{A}}(\theta, \mathbf{b}_X; \hat{\boldsymbol{\beta}}_Y) = \begin{bmatrix}\mathbf{I}_K & \mathbf{A}^{-1}\mathbf{B}\end{bmatrix}(\hat{\boldsymbol{\beta}}_Y - \theta\mathbf{b}_X) = (\hat{\boldsymbol{\beta}}_{Y_{\mathcal{A}}} - \theta\mathbf{b}_{X_{\mathcal{A}}}) + \mathbf{A}^{-1}\mathbf{B}(\hat{\boldsymbol{\beta}}_{Y_{\mathcal{I}}} - \theta\mathbf{b}_{X_{\mathcal{I}}}), \quad (2)$$

$$\mathbf{b}_X(\theta, \mathbf{r}; \hat{\boldsymbol{\beta}}_X, \hat{\boldsymbol{\beta}}_Y, \boldsymbol{\Sigma}_X, \boldsymbol{\Sigma}_Y) = (\theta^2\boldsymbol{\Sigma}_Y^{-1} + \boldsymbol{\Sigma}_X^{-1})^{-1}(\theta\boldsymbol{\Sigma}_Y^{-1}(\hat{\boldsymbol{\beta}}_Y - \mathbf{r}) + \boldsymbol{\Sigma}_X^{-1}\hat{\boldsymbol{\beta}}_X), \quad (3)$$

$$\theta(\mathbf{b}_X, \mathbf{r}; \hat{\boldsymbol{\beta}}_Y, \boldsymbol{\Sigma}_Y) = \frac{(\hat{\boldsymbol{\beta}}_Y^T - \mathbf{r}^T)\boldsymbol{\Sigma}_Y^{-1}\mathbf{b}_X}{\mathbf{b}_X^T\boldsymbol{\Sigma}_Y^{-1}\mathbf{b}_X}, \quad (4)$$

where $\boldsymbol{\Sigma}_Y^{-1} = \begin{bmatrix} \mathbf{A} & \mathbf{B} \\ \mathbf{C} & \mathbf{D} \end{bmatrix}$, $\mathbf{A}$ is a $K \times K$ symmetric matrix, $\mathbf{D}$ is a $(m-K) \times (m-K)$ symmetric matrix, $\mathbf{B}$ is a $K \times (m-K)$ matrix, and $\mathbf{C} = \mathbf{B}^T$.

Accordingly, we will use the following coordinate-descent algorithm to solve $\{\hat{\theta}, \hat{\mathbf{b}}_X, \hat{\mathbf{r}}\} = \arg\max_{\theta, \mathbf{b}_X, \mathbf{r}} l(\theta, \mathbf{b}_X, \mathbf{r})$ under the constraint of $r_i = 0$ if $i \in \mathcal{I}$ in Algorithm 1 in the main text. At $(l+1)$-th iteration,

Step 1: Update $\mathbf{r}_{\mathcal{A}}^{(l+1)} = (\hat{\boldsymbol{\beta}}_{Y_{\mathcal{A}}} - \theta^{(l)}\mathbf{b}_{X_{\mathcal{A}}}^{(l)}) + \mathbf{A}^{-1}\mathbf{B}(\hat{\boldsymbol{\beta}}_{Y_{\mathcal{I}}} - \theta^{(l)}\mathbf{b}_{X_{\mathcal{I}}}^{(l)})$ (Eq. (2)), and $\mathbf{r}_{\mathcal{I}}^{(l+1)} = \mathbf{0}$;

Step 2: Update $\mathbf{b}_X^{(l+1)} = (\theta^{(l)^2}\boldsymbol{\Sigma}_Y^{-1} + \boldsymbol{\Sigma}_X^{-1})^{-1}(\theta^{(l)}\boldsymbol{\Sigma}_Y^{-1}(\hat{\boldsymbol{\beta}}_Y - \mathbf{r}^{(l+1)}) + \boldsymbol{\Sigma}_X^{-1}\hat{\boldsymbol{\beta}}_X)$ (Eq. (3));

Step 3: Update $\theta^{(l+1)} = \frac{(\hat{\boldsymbol{\beta}}_Y^T - \mathbf{r}^{(l+1)^T})\boldsymbol{\Sigma}_Y^{-1}\mathbf{b}_X^{(l+1)}}{\mathbf{b}_X^{(l+1)^T}\boldsymbol{\Sigma}_Y^{-1}\mathbf{b}_X^{(l+1)}}$ (Eq. (4)).

It is noted that at the convergence, only the IVs in $\mathcal{I}$ will contribute to the estimation of θ (and $\mathbf{b}_{X_{\mathcal{I}}}$). To see this, by comparing Eq. (2) and Eq. (4), after some simple matrix algebra, we have $\theta = \hat{\boldsymbol{\beta}}_{Y_{\mathcal{I}}}^T\boldsymbol{\Sigma}_{Y_{\mathcal{I}}}^{-1}\mathbf{b}_{X_{\mathcal{I}}}/\mathbf{b}_{X_{\mathcal{I}}}^T\boldsymbol{\Sigma}_{Y_{\mathcal{I}}}^{-1}\mathbf{b}_{X_{\mathcal{I}}}$. And by comparing Eq. (2) and Eq. (3), we have $\mathbf{b}_{X_{\mathcal{I}}} = (\theta^2\boldsymbol{\Sigma}_{Y_{\mathcal{I}}}^{-1} + \boldsymbol{\Sigma}_{X_{\mathcal{I}}}^{-1})^{-1}(\theta\boldsymbol{\Sigma}_{Y_{\mathcal{I}}}^{-1}(\hat{\boldsymbol{\beta}}_{Y_{\mathcal{I}}}) + \boldsymbol{\Sigma}_{X_{\mathcal{I}}}^{-1}\hat{\boldsymbol{\beta}}_{X_{\mathcal{I}}})$. Therefore, we have the following proposition:

Proposition 1. *Denote* $\{\hat{\theta}, \hat{\mathbf{b}}_X, \hat{\mathbf{r}}\} = \arg\max_{\theta, \mathbf{b}_X, \mathbf{r}} l(\theta, \mathbf{b}_X, \mathbf{r}; \hat{\boldsymbol{\beta}}_X, \hat{\boldsymbol{\beta}}_Y, \boldsymbol{\Sigma}_X, \boldsymbol{\Sigma}_Y)$ *under the constraint of* $\mathbf{r}_{\mathcal{I}} = \mathbf{0}_{m-K}$*, and* $\{\tilde{\theta}, \tilde{\mathbf{b}}_{X_{\mathcal{I}}}\} = \arg\max_{\theta, \mathbf{b}_{X_{\mathcal{I}}}} l(\theta, \mathbf{b}_{X_{\mathcal{I}}}, \mathbf{0}_{m-K}; \hat{\boldsymbol{\beta}}_{X_{\mathcal{I}}}, \hat{\boldsymbol{\beta}}_{Y_{\mathcal{I}}}, \boldsymbol{\Sigma}_{X_{\mathcal{I}}}, \boldsymbol{\Sigma}_{Y_{\mathcal{I}}})$. *Then* $\hat{\theta} = \tilde{\theta}$ *and* $\hat{\mathbf{b}}_{X_{\mathcal{I}}} = \tilde{\mathbf{b}}_{X_{\mathcal{I}}}$.

The estimation and selection consistency (of BIC) with the cMLE developed in Xue et al.[1] can be carried over to the set-up with correlated IVs. Now we state three assumptions used to prove the estimation consistency and asymptotic normality of our proposed cMLE $\hat{\theta}$.

Assumption 1. *(Plurality valid condition.) Suppose that* $\mathcal{A}_0 = \{i : r_i \neq 0\}$ *is the index set of the true invalid IVs with a non-zero horizontal-pleiotropy effect, and* $K_0 = |\mathcal{A}_0|$. *For any* $\mathcal{A} \subseteq \{1, \ldots, m\}$ *and* $|\mathcal{A}| = K_0$*, if* $\mathcal{A} \neq \mathcal{A}_0$*, then there does not exist any constant* $\tilde{\theta} \neq \theta$ *such that* $b_{Yi} = \tilde{\theta} b_{Xi}$ *for all* $i \in \mathcal{A}^C$.

Assumption 2. *The joint effect estimates* $\hat{\boldsymbol{\beta}}_X \sim \mathcal{MVN}(\mathbf{b}_X, \boldsymbol{\Sigma}_X)$ *and* $\hat{\boldsymbol{\beta}}_Y \sim \mathcal{MVN}(\mathbf{b}_Y, \boldsymbol{\Sigma}_Y)$ *with the known covariance matrices* $\boldsymbol{\Sigma}_X$ *and* $\boldsymbol{\Sigma}_Y$.

Assumption 3. *(Orders of the variances and sample sizes.) Let* $N = \min(N_X, N_Y)$*, there exist positive constants* c_1, c_2 *such that* $c_1/N \leq (\mathbf{\Sigma}_X)_{ij} \leq c_2/N$ *and* $c_1/N \leq (\mathbf{\Sigma}_Y)_{ij} \leq c_2/N$ *for* $i = 1, \ldots, m$*,* $j = 1, \ldots, m$*, i.e.,* $\mathbf{\Sigma}_X$ *and* $\mathbf{\Sigma}_Y$ *are* $\Theta(1/N)$*.*

Proposition 2. *Assumption 1 is equivalent to the plurality condition stated in Theorem 1 in Guo et al.*[2]*.*

Proof. Denote $\mathcal{S} = \{i : b_{Xi} \neq 0\}$ be the set of relevant IVs, $\mathcal{V} = \{i : b_{Xi} \neq 0, r_i = 0\} = \mathcal{S} \setminus \mathcal{A}_0$ be the set of valid IVs. The plurality condition in Guo et al.[2] is $|\mathcal{V}| > \max_{c \neq 0} |\{i \in \mathcal{S} : r_i/b_{Xi} = c\}|$. We will use proof by contradiction to show the equivalence between the two conditions. Now, consider two cases:

(1) When all m IVs are relevant, i.e., $\mathcal{S} = \{1, 2, \ldots, m\}$ and $\mathcal{S}^C = \varnothing$, then $\mathcal{V} = \mathcal{A}_0^C$ and $|\mathcal{V}| = m - K_0$.

($\Rightarrow$): If $\exists \mathcal{A} \neq \mathcal{A}_0$, $|\mathcal{A}| = K_0$ and $\tilde{\theta} \neq \theta$ such that $b_{Yi} = \tilde{\theta} b_{Xi}$ for all $i \in \mathcal{A}^C$, then we have $r_i/b_{Xi} = (b_{Yi} - \theta b_{Xi})/b_{Xi} = \tilde{\theta} - \theta$ for $i \in \mathcal{A}^C$, where $|\mathcal{A}^C| = m - K_0$. Therefore, $\max_{c \neq 0} |\{i \in \mathcal{S} : r_i/b_{Xi} = c\}| \geq m - K_0 = |\mathcal{V}|$.

($\Leftarrow$): If $|\mathcal{V}| \leq \max_{c \neq 0} |\{i \in \mathcal{S} : r_i/b_{Xi} = c\}|$, denote c_m be the maximizing c and $\tilde{\mathcal{V}}$ be one subset of $\{i \in \mathcal{S} : r_i/b_{Xi} = c_m\}$ and $|\tilde{\mathcal{V}}| = m - K_0$. Let $\mathcal{A} = (\mathcal{S} \setminus \mathcal{A}_0) \cup (\mathcal{A}_0 \setminus \tilde{\mathcal{V}})$, then $|\mathcal{A}| = (m - K_0) + (K_0 - m + K_0) = K_0$ and $\mathcal{A}^C = (\mathcal{S} \setminus \mathcal{A}_0)^C \cap (\mathcal{A}_0 \setminus \tilde{\mathcal{V}})^C = (\mathcal{S}^C \cup \mathcal{A}_0) \cap (\mathcal{A}_0^C \cup \tilde{\mathcal{V}}) = (\mathcal{A}_0 \cap \mathcal{A}_0^C) \cup (\mathcal{A}_0 \cap \tilde{\mathcal{V}}) = \tilde{\mathcal{V}}$. Therefore, for $i \in \mathcal{A}^C = \tilde{\mathcal{V}}$, we have $b_{Yi} = \theta b_{Xi} + r_i = (\theta + c_m) b_{Xi}$.

(2) When $\mathcal{S}^C \neq \varnothing$ and $|\mathcal{S}| = s_0$, $|\mathcal{S}^C| = m - s_0$, consider two sub-cases:

(2a) When $\mathcal{A}_0 \setminus \mathcal{S} = \varnothing$, i.e. $\mathcal{A}_0 = \{i : r_i \neq 0, b_{Xi} \neq 0\}$ or $\mathcal{A}_0 \subset \mathcal{S}$, then $|\mathcal{V}| = s_0 - K_0$.

($\Rightarrow$): If $\exists \mathcal{A} \neq \mathcal{A}_0$, $|\mathcal{A}| = K_0$ and $\tilde{\theta} \neq \theta$ such that $b_{Yi} = \tilde{\theta} b_{Xi}$ for all $i \in \mathcal{A}^C$, then we have $r_i/b_{Xi} = (b_{Yi} - \theta b_{Xi})/b_{Xi} = \tilde{\theta} - \theta$ for $i \in \mathcal{A}^C \cap \mathcal{S}$. Therefore, $\max_{c \neq 0} |\{i \in \mathcal{S} : r_i/b_{Xi} = c\}| \geq s_0 - K_0 = |\mathcal{V}|$.

($\Leftarrow$): If $|\mathcal{V}| \leq \max_{c \neq 0} |\{i \in \mathcal{S} : r_i/b_{Xi} = c\}|$, denote c_m be the maximizing c and $\tilde{\mathcal{V}}$ be one subset of $\{i \in \mathcal{S} : r_i/b_{Xi} = c_m\}$ and $|\tilde{\mathcal{V}}| = s_0 - K_0$. Let $\mathcal{A} = (\mathcal{S} \setminus \mathcal{A}_0) \cup (\mathcal{A}_0 \setminus \tilde{\mathcal{V}})$, then $|\mathcal{A}| = (s_0 - K_0) + (K_0 - s_0 + K_0) = K_0$ and

$\mathcal{A}^C = (\mathcal{S}\setminus\mathcal{A}_0)^C\cap(\mathcal{A}_0\setminus\tilde{\mathcal{V}})^C = (\mathcal{S}^C\cup\mathcal{A}_0)\cap(\mathcal{A}_0^C\cup\tilde{\mathcal{V}}) = (\mathcal{S}^C\cap\mathcal{A}_0^C)\cup(\mathcal{A}_0\cap\mathcal{A}_0^C)\cup(\mathcal{S}^C\cap\tilde{\mathcal{V}})\cup(\mathcal{A}_0\cap\tilde{\mathcal{V}}) = \mathcal{S}^C\cup\tilde{\mathcal{V}}$. Note that for $i\in\mathcal{S}^C$, $b_{Xi}=0$ and $b_{Yi}=0$. Therefore, we have $b_{Yi}=\theta b_{Xi}+r_i=(\theta+c_m)b_{Xi}$ for $i\in\mathcal{A}^C=\mathcal{S}^C\cup\tilde{\mathcal{V}}$.

(2b) When $\mathcal{A}_0\setminus\mathcal{S}=\{i: r_i\neq 0, b_{Xi}=0\}\neq\varnothing$, further denote $|\mathcal{A}_0\setminus\mathcal{S}|=d_0$, then $|\mathcal{A}_0\cap\mathcal{S}|=K_0-d_0$ and $|\mathcal{V}|=s_0-(K_0-d_0)$.

($\Rightarrow$): If $\exists\mathcal{A}\neq\mathcal{A}_0$, $|\mathcal{A}|=K_0$ and $\tilde{\theta}\neq\theta$ such that $b_{Yi}=\tilde{\theta}b_{Xi}$ for all $i\in\mathcal{A}^C$, then we have $r_i/b_{Xi}=(b_{Yi}-\theta b_{Xi})/b_{Xi}=\tilde{\theta}-\theta$ for $i\in\mathcal{A}^C\cap\mathcal{S}$. We note that since $b_{Yi}=\tilde{\theta}b_{Xi}$ will not hold for $i\in\mathcal{A}_0\setminus\mathcal{S}$, $\mathcal{A}_0\setminus\mathcal{S}$ must be a subset of $\mathcal{A}$. Therefore, $\max_{c\neq 0}|\{i\in\mathcal{S}: r_i/b_{Xi}=c\}|\geq s_0-(K_0-d_0)=|\mathcal{V}|$.

($\Leftarrow$): If $|\mathcal{V}|\leq\max_{c\neq 0}|\{i\in\mathcal{S}: r_i/b_{Xi}=c\}|$, denote c_m be the maximizing c and $\tilde{\mathcal{V}}$ be one subset of $\{i\in\mathcal{S}: r_i/b_{Xi}=c_m\}$ and $|\tilde{\mathcal{V}}|=s_0-K_0+d_0$. Let $\mathcal{A}=(\mathcal{S}\setminus\mathcal{A}_0)\cup(\mathcal{A}_0\setminus\tilde{\mathcal{V}})$, then $|\mathcal{A}|=(s_0-K_0+d_0)+(K_0-s_0+K_0-d_0)=K_0$ and $\mathcal{A}^C=(\mathcal{S}\setminus\mathcal{A}_0)^C\cap(\mathcal{A}_0\setminus\tilde{\mathcal{V}})^C=(\mathcal{S}^C\cup\mathcal{A}_0)\cap(\mathcal{A}_0^C\cup\tilde{\mathcal{V}})=(\mathcal{S}^C\cap\mathcal{A}_0^C)\cup(\mathcal{A}_0\cap\mathcal{A}_0^C)\cup(\mathcal{S}^C\cap\tilde{\mathcal{V}})\cup(\mathcal{A}_0\cap\tilde{\mathcal{V}})=(\mathcal{S}^C\cap\mathcal{A}_0^C)\cup\tilde{\mathcal{V}}$. Note that for $i\in\mathcal{S}^C$, $b_{Xi}=0$ and for $i\in\mathcal{A}_0^C$, $r_i=0$, hence for $i\in(\mathcal{S}^C\cap\mathcal{A}_0^C)$, $b_{Xi}=b_{Yi}=0$. Therefore, we have $b_{Yi}=\theta b_{Xi}+r_i=(\theta+c_m)b_{Xi}$ for $i\in\mathcal{A}^C=(\mathcal{S}^C\cap\mathcal{A}_0^C)\cup\tilde{\mathcal{V}}$.

□

Theorem 1. *With Assumptions 1 to 3 satisfied, if $K_0\in\mathcal{K}$, we have $P(\hat{K}=K_0)\to 1$ and $P(\hat{\mathcal{A}}_{\hat{K}}=\mathcal{A}_0)\to 1$ as $N\to\infty$. And the proposed constrained maximum likelihood estimator $\hat{\theta}$, combined with the use of the BIC selection criterion, is consistent for the true causal effect size θ_0, and*

$$\sqrt{V}(\hat{\theta}-\theta_0)\xrightarrow{d}\mathcal{N}(0,1)\ \textit{as}\ N\to\infty,$$

where $V=\mathbf{b}_{X\mathcal{I}_0}^T(\mathbf{\Sigma}_{Y\mathcal{I}_0}+\theta_0^2\mathbf{\Sigma}_{X\mathcal{I}_0})^{-1}\mathbf{b}_{X\mathcal{I}_0}$ is the expected Fisher information for the profile log-likelihood with IVs in $\mathcal{I}_0$ that can be consistently estimated by its sample version.

To prove Theorem 1, we will first prove the selection consistency of BIC, i.e., it will select the correct set of pleiotropic IVs ($\mathcal{A}_0$) as the sample size N goes to infinity. Then

we prove the estimation consistency and asymptotic normality based on the set of IVs without horizontal pleiotropy.

Lemma 1. *With Assumptions 1-3 satisfied, if* $K_0 \in \mathcal{K}$*, we have* $P(\hat{K} = K_0) \to 1$ *and* $P(\hat{\mathcal{A}}_{\hat{K}} = \mathcal{A}_0) \to 1$ *as* $N \to \infty$.

Proof. First, we show $P(\hat{\mathcal{A}}_{K_0} = \mathcal{A}_0) \to 1$, which is equivalent to show for any $\mathcal{A}_1 \subseteq \{1, \cdots, m\}$ such that $|\mathcal{A}_1| = K_0$ and $\mathcal{A}_1 \neq \mathcal{A}_0$, $P(\hat{\mathcal{A}}_{K_0} = \mathcal{A}_1) \to 0$ as $N \to \infty$. Denote $\mathcal{I}_0 = \mathcal{A}_0^C$, $\mathcal{I}_1 = \mathcal{A}_1^C$, then by Proposition 1, we have

$$
\begin{aligned}
&P(\hat{\mathcal{A}}_{K_0} = \mathcal{A}_1)\\
&\le P\Big\{ \min_{\tilde{\theta}, \tilde{\mathbf{b}}_{X_{\mathcal{I}_1}}} (\hat{\boldsymbol{\beta}}_{X_{\mathcal{I}_1}} - \tilde{\mathbf{b}}_{X_{\mathcal{I}_1}})^T \boldsymbol{\Sigma}_{X_{\mathcal{I}_1}}^{-1} (\hat{\boldsymbol{\beta}}_{X_{\mathcal{I}_1}} - \tilde{\mathbf{b}}_{X_{\mathcal{I}_1}}) + (\hat{\boldsymbol{\beta}}_{Y_{\mathcal{I}_1}} - \tilde{\theta}\tilde{\mathbf{b}}_{X_{\mathcal{I}_1}})^T \boldsymbol{\Sigma}_{Y_{\mathcal{I}_1}}^{-1} (\hat{\boldsymbol{\beta}}_{Y_{\mathcal{I}_1}} - \tilde{\theta}\tilde{\mathbf{b}}_{X_{\mathcal{I}_1}})\\
&\qquad \le (\hat{\boldsymbol{\beta}}_{X_{\mathcal{I}_0}} - \mathbf{b}_{X_{\mathcal{I}_0}})^T \boldsymbol{\Sigma}_{X_{\mathcal{I}_0}}^{-1} (\hat{\boldsymbol{\beta}}_{X_{\mathcal{I}_0}} - \mathbf{b}_{X_{\mathcal{I}_0}}) + (\hat{\boldsymbol{\beta}}_{Y_{\mathcal{I}_0}} - \theta\mathbf{b}_{X_{\mathcal{I}_0}})^T \boldsymbol{\Sigma}_{Y_{\mathcal{I}_0}}^{-1} (\hat{\boldsymbol{\beta}}_{Y_{\mathcal{I}_0}} - \theta\mathbf{b}_{X_{\mathcal{I}_0}})\Big\}.
\end{aligned}
$$

Note that, for $i \in \mathcal{I}_0$, $b_{Yi} = \theta b_{Xi}$, and $\hat{\boldsymbol{\beta}}_{X_{\mathcal{I}_0}} - \mathbf{b}_{X_{\mathcal{I}_0}} \sim \mathcal{MVN}(\mathbf{0}, \boldsymbol{\Sigma}_{X_{\mathcal{I}_0}})$, $\hat{\boldsymbol{\beta}}_{Y_{\mathcal{I}_0}} - \theta\mathbf{b}_{X_{\mathcal{I}_0}} \sim \mathcal{MVN}(\mathbf{0}, \boldsymbol{\Sigma}_{Y_{\mathcal{I}_0}})$. So for any $\epsilon > 0$, as $N \to \infty$, there exists $C > 0$ such that

$$
P\Big\{(\hat{\boldsymbol{\beta}}_{X_{\mathcal{I}_0}} - \mathbf{b}_{X_{\mathcal{I}_0}})^T \boldsymbol{\Sigma}_{X_{\mathcal{I}_0}}^{-1} (\hat{\boldsymbol{\beta}}_{X_{\mathcal{I}_0}} - \mathbf{b}_{X_{\mathcal{I}_0}}) + (\hat{\boldsymbol{\beta}}_{Y_{\mathcal{I}_0}} - \theta\mathbf{b}_{X_{\mathcal{I}_0}})^T \boldsymbol{\Sigma}_{Y_{\mathcal{I}_0}}^{-1} (\hat{\boldsymbol{\beta}}_{Y_{\mathcal{I}_0}} - \theta\mathbf{b}_{X_{\mathcal{I}_0}}) > C\Big\} < \frac{\epsilon}{2}. \tag{5}
$$

And we have

$$
\begin{aligned}
&P\Big\{ \min_{\tilde{\theta}, \tilde{\mathbf{b}}_{X_{\mathcal{I}_1}}} (\hat{\boldsymbol{\beta}}_{X_{\mathcal{I}_1}} - \tilde{\mathbf{b}}_{X_{\mathcal{I}_1}})^T \boldsymbol{\Sigma}_{X_{\mathcal{I}_1}}^{-1} (\hat{\boldsymbol{\beta}}_{X_{\mathcal{I}_1}} - \tilde{\mathbf{b}}_{X_{\mathcal{I}_1}}) + (\hat{\boldsymbol{\beta}}_{Y_{\mathcal{I}_1}} - \tilde{\theta}\tilde{\mathbf{b}}_{X_{\mathcal{I}_1}})^T \boldsymbol{\Sigma}_{Y_{\mathcal{I}_1}}^{-1} (\hat{\boldsymbol{\beta}}_{Y_{\mathcal{I}_1}} - \tilde{\theta}\tilde{\mathbf{b}}_{X_{\mathcal{I}_1}})\\
&\qquad \le (\hat{\boldsymbol{\beta}}_{X_{\mathcal{I}_0}} - \mathbf{b}_{X_{\mathcal{I}_0}})^T \boldsymbol{\Sigma}_{X_{\mathcal{I}_0}}^{-1} (\hat{\boldsymbol{\beta}}_{X_{\mathcal{I}_0}} - \mathbf{b}_{X_{\mathcal{I}_0}}) + (\hat{\boldsymbol{\beta}}_{Y_{\mathcal{I}_0}} - \theta\mathbf{b}_{X_{\mathcal{I}_0}})^T \boldsymbol{\Sigma}_{Y_{\mathcal{I}_0}}^{-1} (\hat{\boldsymbol{\beta}}_{Y_{\mathcal{I}_0}} - \theta\mathbf{b}_{X_{\mathcal{I}_0}})\Big\}\\
&\le P\Big\{ \min_{\tilde{\theta}, \tilde{\mathbf{b}}_{X_{\mathcal{I}_1}}} (\hat{\boldsymbol{\beta}}_{X_{\mathcal{I}_1}} - \tilde{\mathbf{b}}_{X_{\mathcal{I}_1}})^T \boldsymbol{\Sigma}_{X_{\mathcal{I}_1}}^{-1} (\hat{\boldsymbol{\beta}}_{X_{\mathcal{I}_1}} - \tilde{\mathbf{b}}_{X_{\mathcal{I}_1}}) + (\hat{\boldsymbol{\beta}}_{Y_{\mathcal{I}_1}} - \tilde{\theta}\tilde{\mathbf{b}}_{X_{\mathcal{I}_1}})^T \boldsymbol{\Sigma}_{Y_{\mathcal{I}_1}}^{-1} (\hat{\boldsymbol{\beta}}_{Y_{\mathcal{I}_1}} - \tilde{\theta}\tilde{\mathbf{b}}_{X_{\mathcal{I}_1}}) \le C\Big\}\\
&\quad + P\Big\{(\hat{\boldsymbol{\beta}}_{X_{\mathcal{I}_0}} - \mathbf{b}_{X_{\mathcal{I}_0}})^T \boldsymbol{\Sigma}_{X_{\mathcal{I}_0}}^{-1} (\hat{\boldsymbol{\beta}}_{X_{\mathcal{I}_0}} - \mathbf{b}_{X_{\mathcal{I}_0}}) + (\hat{\boldsymbol{\beta}}_{Y_{\mathcal{I}_0}} - \theta\mathbf{b}_{X_{\mathcal{I}_0}})^T \boldsymbol{\Sigma}_{Y_{\mathcal{I}_0}}^{-1} (\hat{\boldsymbol{\beta}}_{Y_{\mathcal{I}_0}} - \theta\mathbf{b}_{X_{\mathcal{I}_0}}) > C\Big\}.
\end{aligned}
$$

After profiling out $\tilde{\mathbf{b}}_{X_{\mathcal{I}_1}}$, we get

$$
\begin{aligned}
&\min_{\tilde{\theta}, \tilde{\mathbf{b}}_{X_{\mathcal{I}_1}}} (\hat{\boldsymbol{\beta}}_{X_{\mathcal{I}_1}} - \tilde{\mathbf{b}}_{X_{\mathcal{I}_1}})^T \boldsymbol{\Sigma}_{X_{\mathcal{I}_1}}^{-1} (\hat{\boldsymbol{\beta}}_{X_{\mathcal{I}_1}} - \tilde{\mathbf{b}}_{X_{\mathcal{I}_1}}) + (\hat{\boldsymbol{\beta}}_{Y_{\mathcal{I}_1}} - \tilde{\theta}\tilde{\mathbf{b}}_{X_{\mathcal{I}_1}})^T \boldsymbol{\Sigma}_{Y_{\mathcal{I}_1}}^{-1} (\hat{\boldsymbol{\beta}}_{Y_{\mathcal{I}_1}} - \tilde{\theta}\tilde{\mathbf{b}}_{X_{\mathcal{I}_1}})\\
&= \min_{\tilde{\theta}} (\hat{\boldsymbol{\beta}}_{Y_{\mathcal{I}_1}} - \tilde{\theta}\hat{\boldsymbol{\beta}}_{X_{\mathcal{I}_1}})^T (\boldsymbol{\Sigma}_{Y_{\mathcal{I}_1}} + \tilde{\theta}^2 \boldsymbol{\Sigma}_{X_{\mathcal{I}_1}})^{-1} (\hat{\boldsymbol{\beta}}_{Y_{\mathcal{I}_1}} - \tilde{\theta}\hat{\boldsymbol{\beta}}_{X_{\mathcal{I}_1}}),
\end{aligned}
$$

so

$$P\Big\{\min_{\tilde{\theta},\tilde{\mathbf{b}}_{X_{\mathcal{I}_1}}}(\hat{\boldsymbol{\beta}}_{X_{\mathcal{I}_1}}-\tilde{\mathbf{b}}_{X_{\mathcal{I}_1}})^T\boldsymbol{\Sigma}_{X_{\mathcal{I}_1}}^{-1}(\hat{\boldsymbol{\beta}}_{X_{\mathcal{I}_1}}-\tilde{\mathbf{b}}_{X_{\mathcal{I}_1}})+(\hat{\boldsymbol{\beta}}_{Y_{\mathcal{I}_1}}-\tilde{\theta}\tilde{\mathbf{b}}_{X_{\mathcal{I}_1}})^T\boldsymbol{\Sigma}_{Y_{\mathcal{I}_1}}^{-1}(\hat{\boldsymbol{\beta}}_{Y_{\mathcal{I}_1}}-\tilde{\theta}\tilde{\mathbf{b}}_{X_{\mathcal{I}_1}})\le C\Big\}$$

$$=P\Big\{\min_{\tilde{\theta}}(\hat{\boldsymbol{\beta}}_{Y_{\mathcal{I}_1}}-\tilde{\theta}\hat{\boldsymbol{\beta}}_{X_{\mathcal{I}_1}})^T(\boldsymbol{\Sigma}_{Y_{\mathcal{I}_1}}+\tilde{\theta}^2\boldsymbol{\Sigma}_{X_{\mathcal{I}_1}})^{-1}(\hat{\boldsymbol{\beta}}_{Y_{\mathcal{I}_1}}-\tilde{\theta}\hat{\boldsymbol{\beta}}_{X_{\mathcal{I}_1}})\le C\Big\}.$$

We have $(\boldsymbol{\Sigma}_{Y_{\mathcal{I}_1}}+\tilde{\theta}^2\boldsymbol{\Sigma}_{X_{\mathcal{I}_1}})^{-1/2}(\hat{\boldsymbol{\beta}}_{Y_{\mathcal{I}_1}}-\tilde{\theta}\hat{\boldsymbol{\beta}}_{X_{\mathcal{I}_1}})\sim\mathcal{MVN}(\boldsymbol{\mu}_{\tilde{\theta}},\mathbf{I}_{m-K_0})$, where $\boldsymbol{\mu}_{\tilde{\theta}}=(\boldsymbol{\Sigma}_{Y_{\mathcal{I}_1}}+\tilde{\theta}^2\boldsymbol{\Sigma}_{X_{\mathcal{I}_1}})^{-1/2}(\theta\mathbf{b}_{X_{\mathcal{I}_1}}+\mathbf{r}_{\mathcal{I}_1}-\tilde{\theta}\mathbf{b}_{X_{\mathcal{I}_1}})$. Hence $(\hat{\boldsymbol{\beta}}_{Y_{\mathcal{I}_1}}-\tilde{\theta}\hat{\boldsymbol{\beta}}_{X_{\mathcal{I}_1}})^T(\boldsymbol{\Sigma}_{Y_{\mathcal{I}_1}}+\tilde{\theta}^2\boldsymbol{\Sigma}_{X_{\mathcal{I}_1}})^{-1}(\hat{\boldsymbol{\beta}}_{Y_{\mathcal{I}_1}}-\tilde{\theta}\hat{\boldsymbol{\beta}}_{X_{\mathcal{I}_1}})$ follows non-central χ^2 distribution with degrees of freedom $(m-K_0)$ and non-centrality parameter $\lambda_{\tilde{\theta}}$ depending on $\tilde{\theta}$:

$$\lambda_{\tilde{\theta}}=(\theta\mathbf{b}_{X_{\mathcal{I}_1}}+\mathbf{r}_{\mathcal{I}_1}-\tilde{\theta}\mathbf{b}_{X_{\mathcal{I}_1}})^T(\boldsymbol{\Sigma}_{Y_{\mathcal{I}_1}}+\tilde{\theta}^2\boldsymbol{\Sigma}_{X_{\mathcal{I}_1}})^{-1}(\theta\mathbf{b}_{X_{\mathcal{I}_1}}+\mathbf{r}_{\mathcal{I}_1}-\tilde{\theta}\mathbf{b}_{X_{\mathcal{I}_1}}).$$

With Assumption 1, there is no $\tilde{\theta}$ making $\theta\cdot b_{Xi}+r_i-\tilde{\theta}\cdot b_{Xi}=0$ for all $i\in\mathcal{A}_1^c$ simultaneously, and with Assumption 3, we have $\min_{\tilde{\theta}}\lambda_{\tilde{\theta}}\to\infty$ as $N\to\infty$. Then as N large enough, we have

$$P\Big\{\min_{\tilde{\theta}}(\hat{\boldsymbol{\beta}}_{Y_{\mathcal{I}_1}}-\tilde{\theta}\hat{\boldsymbol{\beta}}_{X_{\mathcal{I}_1}})^T(\boldsymbol{\Sigma}_{Y_{\mathcal{I}_1}}+\tilde{\theta}^2\boldsymbol{\Sigma}_{X_{\mathcal{I}_1}})^{-1}(\hat{\boldsymbol{\beta}}_{Y_{\mathcal{I}_1}}-\tilde{\theta}\hat{\boldsymbol{\beta}}_{X_{\mathcal{I}_1}})\le C\Big\}\le\frac{\epsilon}{2}. \quad (6)$$

Combining (5) and (6), we get $P(\hat{\mathcal{A}}_{K_0}=\mathcal{A}_0)\to1$ as $N\to\infty$.

Next, we show $P(\hat{K}=K_0)\to1$. For any $K_1<K_0$, we have

$$\begin{aligned}P(\hat{K}=K_1)&\le P\big\{\mathrm{BIC}(K_1)\le\mathrm{BIC}(K_0)\big\}\\&=P\Big\{2l\left(\hat{\theta}(K_0),\hat{\mathbf{b}}_X(K_0),\hat{\mathbf{r}}(K_0)\right)-2l\left(\hat{\theta}(K_1),\hat{\mathbf{b}}_X(K_1),\hat{\mathbf{r}}(K_1)\right)\le\log(N)(K_0-K_1)\Big\}.\end{aligned}$$

As we have shown $P(\hat{\mathcal{A}}_{K_0}=\mathcal{A}_0)\to1$, with probability goes to 1 we have

$$\begin{aligned}&2l\left(\hat{\theta}(K_0),\hat{\mathbf{b}}_X(K_0),\hat{\mathbf{r}}(K_0)\right)-2l\left(\hat{\theta}(K_1),\hat{\mathbf{b}}_X(K_1),\hat{\mathbf{r}}(K_1)\right)\\&\ge\min_{\tilde{\theta},\tilde{\mathbf{b}}_{X_{\mathcal{I}_{K_1}}}}(\hat{\boldsymbol{\beta}}_{X_{\mathcal{I}_{K_1}}}-\tilde{\mathbf{b}}_{X_{\mathcal{I}_{K_1}}})^T\boldsymbol{\Sigma}_{X_{\mathcal{I}_{K_1}}}^{-1}(\hat{\boldsymbol{\beta}}_{X_{\mathcal{I}_{K_1}}}-\tilde{\mathbf{b}}_{X_{\mathcal{I}_{K_1}}})+(\hat{\boldsymbol{\beta}}_{Y_{\mathcal{I}_{K_1}}}-\tilde{\theta}\tilde{\mathbf{b}}_{X_{\mathcal{I}_{K_1}}})^T\boldsymbol{\Sigma}_{Y_{\mathcal{I}_{K_1}}}^{-1}(\hat{\boldsymbol{\beta}}_{Y_{\mathcal{I}_{K_1}}}-\tilde{\theta}\tilde{\mathbf{b}}_{X_{\mathcal{I}_{K_1}}})\\&\quad-(\hat{\boldsymbol{\beta}}_{X_{\mathcal{I}_0}}-\mathbf{b}_{X_{\mathcal{I}_0}})^T\boldsymbol{\Sigma}_{X_{\mathcal{I}_0}}^{-1}(\hat{\boldsymbol{\beta}}_{X_{\mathcal{I}_0}}-\mathbf{b}_{X_{\mathcal{I}_0}})+(\hat{\boldsymbol{\beta}}_{Y_{\mathcal{I}_0}}-\theta\mathbf{b}_{X_{\mathcal{I}_0}})^T\boldsymbol{\Sigma}_{Y_{\mathcal{I}_0}}^{-1}(\hat{\boldsymbol{\beta}}_{Y_{\mathcal{I}_0}}-\theta\mathbf{b}_{X_{\mathcal{I}_0}}).\end{aligned}$$

Then we get

$$P(\hat{K}=K_1) \leq \sum_{|\mathcal{I}|=m-K_1} P\Big\{ \min_{\tilde{\theta},\tilde{\mathbf{b}}_{X_\mathcal{I}}} (\hat{\boldsymbol{\beta}}_{X_\mathcal{I}} - \tilde{\mathbf{b}}_{X_\mathcal{I}})^T \boldsymbol{\Sigma}_{X_\mathcal{I}}^{-1} (\hat{\boldsymbol{\beta}}_{X_\mathcal{I}} - \tilde{\mathbf{b}}_{X_\mathcal{I}}) + (\hat{\boldsymbol{\beta}}_{Y_\mathcal{I}} - \tilde{\theta}\tilde{\mathbf{b}}_{X_\mathcal{I}})^T \boldsymbol{\Sigma}_{Y_\mathcal{I}}^{-1} (\hat{\boldsymbol{\beta}}_{Y_\mathcal{I}} - \tilde{\theta}\tilde{\mathbf{b}}_{X_\mathcal{I}})$$

$$\leq (\hat{\boldsymbol{\beta}}_{X_{\mathcal{I}_0}} - \mathbf{b}_{X_{\mathcal{I}_0}})^T \boldsymbol{\Sigma}_{X_{\mathcal{I}_0}}^{-1} (\hat{\boldsymbol{\beta}}_{X_{\mathcal{I}_0}} - \mathbf{b}_{X_{\mathcal{I}_0}}) + (\hat{\boldsymbol{\beta}}_{Y_{\mathcal{I}_0}} - \theta \mathbf{b}_{X_{\mathcal{I}_0}})^T \boldsymbol{\Sigma}_{Y_{\mathcal{I}_0}}^{-1} (\hat{\boldsymbol{\beta}}_{Y_{\mathcal{I}_0}} - \theta \mathbf{b}_{X_{\mathcal{I}_0}}) + \log(N)(K_0 - K_1) \Big\}.$$

Similar to above, we get

$$\begin{aligned} &\min_{\tilde{\theta},\tilde{\mathbf{b}}_{X_\mathcal{I}}} (\hat{\boldsymbol{\beta}}_{X_\mathcal{I}} - \tilde{\mathbf{b}}_{X_{\mathcal{I}_1}})^T \boldsymbol{\Sigma}_{X_\mathcal{I}}^{-1} (\hat{\boldsymbol{\beta}}_{X_\mathcal{I}} - \tilde{\mathbf{b}}_{X_\mathcal{I}}) + (\hat{\boldsymbol{\beta}}_{Y_\mathcal{I}} - \tilde{\theta}\tilde{\mathbf{b}}_{X_\mathcal{I}})^T \boldsymbol{\Sigma}_{Y_\mathcal{I}}^{-1} (\hat{\boldsymbol{\beta}}_{Y_\mathcal{I}} - \tilde{\theta}\tilde{\mathbf{b}}_{X_\mathcal{I}}) \\ &= \min_{\tilde{\theta}} (\hat{\boldsymbol{\beta}}_{Y_\mathcal{I}} - \tilde{\theta}\hat{\boldsymbol{\beta}}_{X_\mathcal{I}})^T (\boldsymbol{\Sigma}_{Y_\mathcal{I}} + \tilde{\theta}^2 \boldsymbol{\Sigma}_{X_\mathcal{I}})^{-1} (\hat{\boldsymbol{\beta}}_{Y_\mathcal{I}} - \tilde{\theta}\hat{\boldsymbol{\beta}}_{X_\mathcal{I}}), \end{aligned}$$

and $(\hat{\boldsymbol{\beta}}_{Y_\mathcal{I}} - \tilde{\theta}\hat{\boldsymbol{\beta}}_{X_\mathcal{I}})^T (\boldsymbol{\Sigma}_{Y_\mathcal{I}} + \tilde{\theta}^2 \boldsymbol{\Sigma}_{X_\mathcal{I}})^{-1} (\hat{\boldsymbol{\beta}}_{Y_\mathcal{I}} - \tilde{\theta}\hat{\boldsymbol{\beta}}_{X_\mathcal{I}})$ follows non-central χ^2 distribution with degrees of freedom $(m-K_1)$ and non-centrality parameter $\lambda_{\tilde{\theta}}$. Similarly, since $K_1 < K_0$, and with Assumptions 1 and 3, we have for any $|\mathcal{A}| = K_1$,

$$\begin{aligned} &P\Big\{ \min_{\tilde{\theta},\tilde{\mathbf{b}}_{X_\mathcal{I}}} (\hat{\boldsymbol{\beta}}_{X_\mathcal{I}} - \tilde{\mathbf{b}}_{X_\mathcal{I}})^T \boldsymbol{\Sigma}_{X_\mathcal{I}}^{-1} (\hat{\boldsymbol{\beta}}_{X_\mathcal{I}} - \tilde{\mathbf{b}}_{X_\mathcal{I}}) + (\hat{\boldsymbol{\beta}}_{Y_\mathcal{I}} - \tilde{\theta}\tilde{\mathbf{b}}_{X_\mathcal{I}})^T \boldsymbol{\Sigma}_{Y_\mathcal{I}}^{-1} (\hat{\boldsymbol{\beta}}_{Y_\mathcal{I}} - \tilde{\theta}\tilde{\mathbf{b}}_{X_\mathcal{I}}) \\ &\leq (\hat{\boldsymbol{\beta}}_{X_{\mathcal{I}_0}} - \mathbf{b}_{X_{\mathcal{I}_0}})^T \boldsymbol{\Sigma}_{X_{\mathcal{I}_0}}^{-1} (\hat{\boldsymbol{\beta}}_{X_{\mathcal{I}_0}} - \mathbf{b}_{X_{\mathcal{I}_0}}) + (\hat{\boldsymbol{\beta}}_{Y_{\mathcal{I}_0}} - \theta \mathbf{b}_{X_{\mathcal{I}_0}})^T \boldsymbol{\Sigma}_{Y_{\mathcal{I}_0}}^{-1} (\hat{\boldsymbol{\beta}}_{Y_{\mathcal{I}_0}} - \theta \mathbf{b}_{X_{\mathcal{I}_0}}) \\ &\qquad + \log(N)(K_0 - K_1) \Big\} \to 0, \ as\ N \to \infty. \end{aligned}$$

This gives us $P(\hat{K} = K_1) \to 0$ for any $K_1 < K_0$. For any $K_1 > K_0$, we have

$$\begin{aligned} P(\hat{K}=K_1) \leq P\Big\{ &\log(N)(K_1 - K_0) \leq \\ &(\hat{\boldsymbol{\beta}}_{X_{\mathcal{I}_0}} - \mathbf{b}_{X_{\mathcal{I}_0}})^T \boldsymbol{\Sigma}_{X_{\mathcal{I}_0}}^{-1} (\hat{\boldsymbol{\beta}}_{X_{\mathcal{I}_0}} - \mathbf{b}_{X_{\mathcal{I}_0}}) + (\hat{\boldsymbol{\beta}}_{Y_{\mathcal{I}_0}} - \theta \mathbf{b}_{X_{\mathcal{I}_0}})^T \boldsymbol{\Sigma}_{Y_{\mathcal{I}_0}}^{-1} (\hat{\boldsymbol{\beta}}_{Y_{\mathcal{I}_0}} - \theta \mathbf{b}_{X_{\mathcal{I}_0}}) \Big\} \end{aligned}$$

Since $(\hat{\boldsymbol{\beta}}_{X_{\mathcal{I}_0}} - \mathbf{b}_{X_{\mathcal{I}_0}})^T \boldsymbol{\Sigma}_{X_{\mathcal{I}_0}}^{-1} (\hat{\boldsymbol{\beta}}_{X_{\mathcal{I}_0}} - \mathbf{b}_{X_{\mathcal{I}_0}}) + (\hat{\boldsymbol{\beta}}_{Y_{\mathcal{I}_0}} - \theta \mathbf{b}_{X_{\mathcal{I}_0}})^T \boldsymbol{\Sigma}_{Y_{\mathcal{I}_0}}^{-1} (\hat{\boldsymbol{\beta}}_{Y_{\mathcal{I}_0}} - \theta \mathbf{b}_{X_{\mathcal{I}_0}})$ is a central chi-square with degrees of freedom $2(m - K_0)$, we get $P(\hat{K} = K_1) \to 0$ for any $K_1 > K_0$. So we have $P(\hat{K} = K_0) \to 1$ as $N \to \infty$. □

Suppose we have correctly selected IVs in $\mathcal{A}_0$, then the resulted cMLE of θ is the same as the maximum profile likelihood estimator (MPLE) (profiling out $\mathbf{b}_{X_{\mathcal{I}_0}}$) based on IVs in $\mathcal{I}_0$. And we will prove the consistency and asymptotic normality based on the MPLE.

For the simplicity of notation, we assume that all m IVs have $r_i = 0$ from now on, i.e.,

$$\begin{aligned}\hat{\boldsymbol{\beta}}_X &\sim \mathcal{MVN}(\mathbf{b}_X, \boldsymbol{\Sigma}_X),\\ \hat{\boldsymbol{\beta}}_Y &\sim \mathcal{MVN}(\theta\mathbf{b}_X, \boldsymbol{\Sigma}_Y).\end{aligned} \tag{7}$$

Then the profile log-likelihood is

$$\tilde{l}(\theta) = -\frac{1}{2}(\hat{\boldsymbol{\beta}}_Y - \theta\hat{\boldsymbol{\beta}}_X)^T(\boldsymbol{\Sigma}_Y + \theta^2\boldsymbol{\Sigma}_X)^{-1}(\hat{\boldsymbol{\beta}}_Y - \theta\hat{\boldsymbol{\beta}}_X). \tag{8}$$

The maximum likelihood estimator of θ is given by $\hat{\theta} = \arg\max_\theta \tilde{l}(\theta)$.

Lemma 2. *Under model Eq.* (7) *and Assumptions 1-3, the maximum likelihood estimator* $\hat{\theta}$ *of Eq.* (8) *is consistent for the true causal parameter* θ_0*, that is,* $\hat{\theta} \xrightarrow{p} \theta_0$ *as* $N \to \infty$*.*

Proof. Let $\mathbf{e} = \hat{\boldsymbol{\beta}}_Y - \mathbf{b}_Y$, $\boldsymbol{\epsilon} = \hat{\boldsymbol{\beta}}_X - \mathbf{b}_X$, $\mathbf{b}_Y = \theta_0\mathbf{b}_X$. After some algebra, we have

$$\begin{aligned}-2\tilde{l}(\theta) =&(\mathbf{e} - \theta\boldsymbol{\epsilon})^T(\boldsymbol{\Sigma}_Y + \theta^2\boldsymbol{\Sigma}_X)^{-1}(\mathbf{e} - \theta\boldsymbol{\epsilon}) +\\ &(\theta_0 - \theta)^2\mathbf{b}_X^T(\boldsymbol{\Sigma}_Y + \theta^2\boldsymbol{\Sigma}_X)^{-1}\mathbf{b}_X + 2(\theta_0 - \theta)\mathbf{b}_X^T(\boldsymbol{\Sigma}_Y + \theta^2\boldsymbol{\Sigma}_X)^{-1}(\mathbf{e} - \theta\boldsymbol{\epsilon}),\\ -2\tilde{l}(\theta_0) =&(\mathbf{e} - \theta_0\boldsymbol{\epsilon})^T(\boldsymbol{\Sigma}_Y + \theta_0^2\boldsymbol{\Sigma}_X)^{-1}(\mathbf{e} - \theta_0\boldsymbol{\epsilon}).\end{aligned}$$

Notice that $(\mathbf{e}-\theta\boldsymbol{\epsilon})^T(\boldsymbol{\Sigma}_Y+\theta^2\boldsymbol{\Sigma}_X)^{-1}(\mathbf{e}-\theta\boldsymbol{\epsilon})$ and $(\mathbf{e}-\theta_0\boldsymbol{\epsilon})^T(\boldsymbol{\Sigma}_Y+\theta_0^2\boldsymbol{\Sigma}_X)^{-1}(\mathbf{e}-\theta_0\boldsymbol{\epsilon})$ follow a χ^2 with degrees of freedom m, and they are both $O_p(1)$ as we consider a fixed number of IVs. Furthermore, given $||\mathbf{b}_X||_2^2$ is bounded and $\mathbf{b}_X \neq \mathbf{0}$, and by Assumption 3, we have for any $\epsilon > 0$, when $|\theta-\theta_0| \geq \epsilon$, $(\theta_0-\theta)^2\mathbf{b}_X^T(\boldsymbol{\Sigma}_Y+\theta^2\boldsymbol{\Sigma}_X)^{-1}\mathbf{b}_X$ is $\Theta(N)$, and $(\theta_0-\theta)\mathbf{b}_X^T(\boldsymbol{\Sigma}_Y+\theta^2\boldsymbol{\Sigma}_X)^{-1}(\mathbf{e}-\theta\boldsymbol{\epsilon})$ is $O_p(\sqrt{N})$. Therefore, we have $P\left(\tilde{l}(\theta_0) > \sup_{|\theta-\theta_0|\geq\epsilon}\tilde{l}(\theta)\right) \to 1$ as $N \to \infty$. □

Next, we study the asymptotic normality of $\hat{\theta}$. Define the profile score to be the derivative of the profile log-likelihood:

$$\begin{aligned}\psi(\theta) := \tilde{l}'(\theta) = \theta(\hat{\boldsymbol{\beta}}_Y - \theta\hat{\boldsymbol{\beta}}_X)^T(\boldsymbol{\Sigma}_Y + \theta^2\boldsymbol{\Sigma}_X)^{-1}\boldsymbol{\Sigma}_X(\boldsymbol{\Sigma}_Y + \theta^2\boldsymbol{\Sigma}_X)^{-1}(\hat{\boldsymbol{\beta}}_Y - \theta\hat{\boldsymbol{\beta}}_X)&\\ +(\hat{\boldsymbol{\beta}}_Y - \theta\hat{\boldsymbol{\beta}}_X)^T(\boldsymbol{\Sigma}_Y + \theta^2\boldsymbol{\Sigma}_X)^{-1}\hat{\boldsymbol{\beta}}_X.&\end{aligned} \tag{9}$$

The Taylor expansion of $\psi(\hat{\theta})$ around the truth θ_0 can be expressed as:

$$0 = \psi(\hat{\theta}) = \psi(\theta_0) + \psi'(\theta_0)(\hat{\theta} - \theta_0) + \frac{1}{2}\psi''(\tilde{\theta})(\hat{\theta} - \theta_0)^2,$$

where $\tilde{\theta}$ is between $\hat{\theta}$ and θ_0. Then we have

$$\sqrt{V}(\hat{\theta} - \theta_0) = \frac{-\psi(\theta_0)/\sqrt{V}}{\psi'(\theta_0)/V + (1/2)\psi''(\tilde{\theta})(\hat{\theta} - \theta_0)/V}, \tag{10}$$

where

$$V = \mathbb{E}[-\psi'(\theta_0)] = \mathbf{b}_X^T(\mathbf{\Sigma}_Y + \theta_0^2\mathbf{\Sigma}_X)^{-1}\mathbf{b}_X. \tag{11}$$

The nominator of Eq. (10) can be proved to converge in distribution to $\mathcal{N}(0, 1)$, the first term in the denominator of Eq. (10) can be proved to converge in probability to 1 and the second term in the denominator can be proved to be negligible given $\hat{\theta}$ is consistent. We first prove these three statements, and by Slutsky's Theorem, the following asymptotic normality of $\hat{\theta}$ can be established.

Lemma 3. *Under the assumptions in Lemma 2, as $N \to \infty$, we have*

$$\sqrt{V}(\hat{\theta} - \theta_0) \xrightarrow{d} \mathcal{N}(0, 1),$$

where $V = \mathbf{b}_X^T(\mathbf{\Sigma}_Y + \theta_0^2\mathbf{\Sigma}_X)^{-1}\mathbf{b}_X$.

Proof. We first show $(1/\sqrt{V})\psi(\theta_0) \xrightarrow{d} \mathcal{N}(0, 1)$.

Denote $\mathbf{e} = \hat{\boldsymbol{\beta}}_Y - \mathbf{b}_Y$, $\boldsymbol{\epsilon} = \hat{\boldsymbol{\beta}}_X - \mathbf{b}_X$, $\mathbf{U}(\theta) = (\mathbf{\Sigma}_Y + \theta^2\mathbf{\Sigma}_X)^{-1}$, $\mathbf{W}(\theta) = -U'(\theta)/(2\theta) = (\mathbf{\Sigma}_Y + \theta^2\mathbf{\Sigma}_X)^{-1}\mathbf{\Sigma}_X(\mathbf{\Sigma}_Y + \theta^2\mathbf{\Sigma}_X)^{-1}$, after some algebra, we can rewrite Eq. (9) to obtain

$$\psi(\theta_0) = \mathbf{b}_X^T\mathbf{U}(\theta_0)(\mathbf{e} - \theta_0\boldsymbol{\epsilon}) + \left[\theta_0(\mathbf{e} - \theta_0\boldsymbol{\epsilon})^T\mathbf{W}(\theta_0)(\mathbf{e} - \theta_0\boldsymbol{\epsilon}) + (\mathbf{e} - \theta_0\boldsymbol{\epsilon})^T\mathbf{U}(\theta_0)\boldsymbol{\epsilon}\right] \tag{12}$$

Since $\mathbf{e} - \theta_0\boldsymbol{\epsilon} \sim \mathcal{MVN}(\mathbf{0}, \mathbf{\Sigma}_Y + \theta_0^2\mathbf{\Sigma}_X)$, the first term on the right hand side in Eq. (12) is distributed as $\mathcal{N}(0, V)$ and $V = \Theta(N||\mathbf{b}_X||_2^2)$. The second term is negligible compared to the first term since it has variance $O(m)$. Therefore, $(1/\sqrt{V})\psi(\theta_0) \to \mathcal{N}(0, 1)$.

We next show $(-1/V)\psi'(\theta_0) \xrightarrow{p} 1$.

Since $\mathbb{E}[-\psi'(\theta_0)] = V$ by definition, it suffices to show $\mathrm{Var}(\psi'(\theta_0)/V) \to 0$. Rewrite Eq. (9) using $\mathbf{e}$, $\boldsymbol{\epsilon}$, $\mathbf{U}(\theta)$ and $\mathbf{W}(\theta)$, we have

$$\psi(\theta) = \theta(\mathbf{e}-\theta\boldsymbol{\epsilon})^T\mathbf{W}(\theta)(\mathbf{e}-\theta\boldsymbol{\epsilon}) + \theta(\theta_0-\theta)^2\mathbf{b}_X^T\mathbf{W}(\theta)\mathbf{b}_X + 2\theta(\theta_0-\theta)(\mathbf{e}-\theta\boldsymbol{\epsilon})^T\mathbf{W}(\theta)\mathbf{b}_X + (\mathbf{e}-\theta\boldsymbol{\epsilon})^T\mathbf{U}(\theta)\boldsymbol{\epsilon} + (\theta_0-\theta)\mathbf{b}_X^T\mathbf{U}(\theta)\boldsymbol{\epsilon} + \mathbf{b}_X^T\mathbf{U}(\theta)(\mathbf{e}-\theta\boldsymbol{\epsilon}) + (\theta_0-\theta)\mathbf{b}_X^T\mathbf{U}(\theta)\mathbf{b}_X. \tag{13}$$

Notice that $\psi(\theta)$ (Eq. (13)) is a homogeneous quadratic polynomial of $(\tilde{\mathbf{b}}_X, \tilde{\mathbf{e}}, \tilde{\boldsymbol{\epsilon}}) = (\sqrt{N}\mathbf{b}_X, \sqrt{N}\mathbf{e}, \sqrt{N}\boldsymbol{\epsilon})$, it is easy to see that $\psi'(\theta)$ is also a homogeneous quadratic polynomial of $(\tilde{\mathbf{b}}_X, \tilde{\mathbf{e}}, \tilde{\boldsymbol{\epsilon}})$. Also given that $\tilde{\mathbf{b}}_X$ is treated as fixed, $\tilde{e}_i \perp\!\!\!\perp \tilde{\epsilon}_j$ and other terms such as $\mathrm{Cov}(\tilde{\mathbf{e}})$, $\mathrm{Cov}(\tilde{\boldsymbol{\epsilon}})$, $\mathrm{Var}(\tilde{\mathbf{e}}^T\tilde{\boldsymbol{\epsilon}})$ are all $O(1)$, we have $\mathrm{Var}(\psi'(\theta)) = O(N||\mathbf{b}_X||_2^2)$ for any θ in a neighbourhood $\mathcal{N}$ of θ_0. Then it is easy to see that $\mathrm{Var}(\psi'(\theta_0)) \ll V^2 = \Theta(N^2||\mathbf{b}_X||^4)$.

Since $\psi'(\theta)$ is a homogeneous quadratic polynomial of $(\tilde{\mathbf{b}}_X, \tilde{\mathbf{e}}, \tilde{\boldsymbol{\epsilon}})$, so is $\psi''(\theta)$, and we can apply the same argument for $\psi'(\theta_0)$ above to $\psi''(\theta)$ and obtain that for a neighborhood $\mathcal{N}$ of θ_0, $\sup_{\theta\in\mathcal{N}}(1/V)\psi''(\theta) = O_p(1)$ and $\psi''(\tilde{\theta})(\hat{\theta}-\theta_0)/V = o_p(1)$

Lastly by Slutsky's Theorem, we have $\sqrt{V}(\hat{\theta}-\theta_0) \xrightarrow{d} \mathcal{N}(0,1)$. □

With Lemmas 1-3, we can conclude Theorem 1. Here are a few points we would like to highlight. Our proposed method can only consistently select the invalid IVs with $r_i \neq 0$ (violating the second and the third IV assumptions stated in the main text), but it cannot select the invalid IVs only violating the first IV assumption, i.e. $b_{Xi} = r_i = 0$. On one hand, the first IV assumption can be tested empirically and is less prone to be violated by using IVs jointly associated with the exposure. On the other hand, even though we mistakenly include some irrelevant IVs in cisMR-cML, it will not affect the validity of the statistical inference for the causal parameter. This can be seen from the proof of Lemmas 2 and 3 that, as long as there are valid IVs such that $\mathbf{b}_X \neq \mathbf{0}$, having a few zero elements in $\mathbf{b}_X$ will not affect the result. Therefore, the proposed method is robust to the violation of any or all of the three IV assumptions under the plurality valid condition. Additionally, it's worth noting that we assume the strength of direct effect r_i does not

depend on the GWAS sample size N (i.e., r_i is $O(1)$). More generally, the proposed BIC can consistently select invalid IVs (Lemma 1) as long as r_i does not diminish with the sample size at a rate equal to or faster than $1/\sqrt{N}$. For example, in Table S16 where $r_i = \kappa/\sqrt[3]{N}$, cisMR-cML-BIC consistently selected invalid IVs as N increased, and yielded correct inference. But in Table S17 where $r_i = \kappa/\sqrt{N}$, the selection performance did not improve even as N increased, and the coverage (based on asymptotic inference) was anti-conservative. In Table S18 where $r_i = \kappa/N$, cisMR-cML-BIC rarely selected any invalid IVs; nevertheless, including such weak invalid IVs did not significantly affect the inference.

S2 LASSO implementation of cisMR-cML

Instead of solving the constrained maximum likelihood proposed in Eq. (11) in the main text by constraining the number of invalid IVs, we also consider imposing a LASSO penalty on the pleiotropic effect r_i as follows:

$$\arg\min_{\theta,\mathbf{b}_X,\mathbf{r}}(\hat{\boldsymbol{\beta}}_X - \mathbf{b}_X)^T\boldsymbol{\Sigma}_X^{-1}(\hat{\boldsymbol{\beta}}_X - \mathbf{b}_X) + (\hat{\boldsymbol{\beta}}_Y - \theta\mathbf{b}_X - \mathbf{r})^T\boldsymbol{\Sigma}_Y^{-1}(\hat{\boldsymbol{\beta}}_Y - \theta\mathbf{b}_X - \mathbf{r}) + \lambda\sum_{i=1}^{m}|r_i|.$$

If r_i is (or is estimated to be) zero, the i-th genetic variant is regarded as a valid IV with no pleiotropic effect. $\lambda > 0$ is a tuning parameter that controls the level of penalization. The larger the value, the fewer IVs will be selected as invalid. For a given $\lambda > 0$, we use a coordinate-descent algorithm to solve the above penalized log-likelihood problem. Starting with the initial value $\theta^{(0)}$ and $\mathbf{b}_X^{(0)}$, repeat for $l = 1, 2, 3, \ldots$:

Step 1: Update $\mathbf{r}^{(l)} = \arg\min_{\mathbf{r}}(\hat{\boldsymbol{\beta}}_Y - \theta^{(l-1)}\mathbf{b}_X^{(l-1)} - \mathbf{r})^T\boldsymbol{\Sigma}_Y^{-1}(\hat{\boldsymbol{\beta}}_Y - \theta^{(l-1)}\mathbf{b}_X^{(l-1)} - \mathbf{r}) + \lambda\sum_{i=1}^{m}|r_i|$;

Step 2: Update $\mathbf{b}_X^{(l)} = (\theta^{(l-1)^2}\boldsymbol{\Sigma}_Y^{-1} + \boldsymbol{\Sigma}_X^{-1})^{-1}(\theta^{(l-1)}\boldsymbol{\Sigma}_Y^{-1}(\hat{\boldsymbol{\beta}}_Y - \mathbf{r}^{(l)}) + \boldsymbol{\Sigma}_X^{-1}\hat{\boldsymbol{\beta}}_X)$;

Step 3: Update $\theta^{(l)} = \frac{(\hat{\boldsymbol{\beta}}_Y^T - \mathbf{r}^{(l)^T})\boldsymbol{\Sigma}_Y^{-1}\mathbf{b}_X^{(l)}}{\mathbf{b}_X^{(l)^T}\boldsymbol{\Sigma}_Y^{-1}\mathbf{b}_X^{(l)}}$.

For Step 1, we can transform it into a standard LASSO problem. Let $\mathbf{\Sigma}_Y^{-1} = \mathbf{L}^T\mathbf{L}$ be the Cholesky decomposition of $\mathbf{\Sigma}_Y^{-1}$, then

$$\text{Step 1: } \mathbf{r}^{(l)} = \arg\min_{\mathbf{r}} \|\mathbf{L}(\hat{\boldsymbol{\beta}}_Y - \theta^{(l-1)}\mathbf{b}_X^{(l-1)}) - \mathbf{L}\mathbf{r}\|_2^2 + \lambda \sum_{i=1}^{m} |r_i|,$$

for which we can solve using standard software (e.g. `glmnet` R package) with responses $\mathbf{L}(\hat{\boldsymbol{\beta}}_Y - \theta^{(l-1)}\mathbf{b}_X^{(l-1)})$ and design matrix $\mathbf{L}$. Once we have selected invalid IVs with non-zero r_i, we perform post-selection estimation using the set of selected valid IVs $\mathcal{I}$ with $|\mathcal{I}| = m - K$:

$$\{\hat{\theta}, \hat{\mathbf{b}}_{X_\mathcal{I}}\} = \arg\max_{\theta, \mathbf{b}_{X_\mathcal{I}}} l(\theta, \mathbf{b}_{X_\mathcal{I}}, \mathbf{0}_{m-K}; \hat{\boldsymbol{\beta}}_{X_\mathcal{I}}, \hat{\boldsymbol{\beta}}_{Y_\mathcal{I}}, \mathbf{\Sigma}_{X_\mathcal{I}}, \mathbf{\Sigma}_{Y_\mathcal{I}}),$$

where l is the log-likelihood defined in Eq. (1). Following the independent-IV version of MR-Lasso[3], we search λ from $(0.1, 0.2, ..., 4.9, 5.0, 5.2, 5.4, ..., 9.8, 10.0)$ by default. Finally, similar to the proposed implementation of cisMR-cML, we use BIC to select the best tuning parameter λ, and perform data perturbation to account for the uncertainty in model selection.

S3 Additional results in the first set of simulations

S3.1 Full results for scenario 1

	$\theta = 0$						$\theta = 0.2$					
Method	TIE	Mean	SD	SE	Cov	RMSE	Power	Mean	SD	SE	Cov	RMSE
$\rho = 0.2$ in the autoregressive LD structure												
cisMR-cML-BIC	0.056	0.000	0.020	0.020	0.944	0.020	1.000	0.200	0.022	0.021	0.948	0.022
**cisMR-cML**	0.020	-0.001	0.020	0.026	0.980	0.020	0.998	0.200	0.022	0.028	0.978	0.022
cML-IND	0.032	0.001	0.036	0.040	0.968	0.036	0.960	0.199	0.041	0.047	0.964	0.041
GEgger	0.034	-0.001	0.035	0.037	0.966	0.035	0.992	0.197	0.038	0.039	0.940	0.038
Egger-IND	0.030	0.006	0.109	0.088	0.970	0.109	0.688	0.204	0.099	0.092	0.956	0.099
GIVW	0.038	0.000	0.019	0.021	0.962	0.019	1.000	0.197	0.021	0.022	0.958	0.021
IVW-IND	0.030	0.001	0.034	0.038	0.970	0.034	0.962	0.197	0.040	0.040	0.936	0.041
LEgger	0.056	-0.001	0.022	0.021	0.972	0.022	1.000	0.197	0.023	0.023	0.940	0.024
MR.LDP	0.030	0.000	0.019	NA	NA	0.019	1.000	0.200	0.021	NA	NA	0.021
MRAID	0.036	-0.001	0.021	NA	NA	0.021	0.994	0.198	0.022	NA	NA	0.022
MR.Corr2	0.046	0.000	0.019	0.020	0.954	0.019	1.000	0.090	0.093	0.010	0.464	0.144
MR.CUE	0.028	0.001	0.019	0.022	0.972	0.019	1.000	0.203	0.022	0.024	0.970	0.022
$\rho = 0.6$ in the autoregressive LD structure												
cisMR-cML-BIC	0.052	0.000	0.020	0.020	0.948	0.020	1.000	0.200	0.022	0.021	0.952	0.022
**cisMR-cML**	0.012	-0.001	0.020	0.030	0.988	0.020	0.998	0.200	0.023	0.032	0.984	0.023
GEgger	0.040	-0.001	0.030	0.031	0.960	0.030	0.996	0.195	0.033	0.033	0.944	0.033
GIVW	0.038	0.000	0.019	0.021	0.962	0.019	1.000	0.197	0.021	0.022	0.952	0.021
IVW-IND	0.042	0.004	0.064	0.048	0.958	0.064	0.942	0.202	0.068	0.048	0.932	0.068
LEgger	0.052	0.000	0.025	0.023	0.962	0.025	0.994	0.196	0.027	0.026	0.948	0.028
MR.LDP	0.030	-0.001	0.019	NA	NA	0.019	1.000	0.200	0.022	NA	NA	0.022
MRAID	0.034	0.000	0.019	NA	NA	0.019	0.998	0.199	0.022	NA	NA	0.022
MR.Corr2	0.050	-0.001	0.019	0.020	0.950	0.019	0.991	0.079	0.091	0.009	0.408	0.152
MR.CUE	0.026	0.001	0.019	0.024	0.974	0.019	1.000	0.203	0.022	0.026	0.976	0.022
$\rho = 0.8$ in the autoregressive LD structure												
cisMR-cML-BIC	0.038	0.000	0.020	0.020	0.962	0.020	1.000	0.200	0.023	0.021	0.954	0.022
**cisMR-cML**	0.006	-0.001	0.022	0.037	0.994	0.022	0.994	0.200	0.024	0.038	0.994	0.024
GEgger	0.044	-0.001	0.032	0.032	0.956	0.032	0.988	0.194	0.035	0.033	0.940	0.035
GIVW	0.024	-0.001	0.018	0.021	0.976	0.018	1.000	0.196	0.021	0.022	0.946	0.021
IVW-IND	0.040	0.036	0.876	0.206	0.960	0.876	0.774	0.199	0.668	0.206	0.934	0.667
LEgger	0.052	0.000	0.033	0.028	0.916	0.033	0.970	0.193	0.035	0.031	0.914	0.036
MR.LDP	0.018	-0.001	0.019	NA	NA	0.019	1.000	0.200	0.021	NA	NA	0.021
MRAID	0.016	-0.001	0.019	NA	NA	0.019	0.996	0.198	0.023	NA	NA	0.023
MR.Corr2	0.028	-0.001	0.019	0.020	0.972	0.019	0.992	0.047	0.080	0.006	0.244	0.173
MR.CUE	0.008	0.000	0.020	0.027	0.992	0.020	0.994	0.201	0.027	0.030	0.988	0.027

Table S1. **Simulation results in scenario 1, where all 10 IVs have an effect on the exposure, and no invalid IV ($K_1 = 0$).** Type-I error (T1E) or power, mean and standard deviation (SD) of estimates, mean standard error (SE) and coverage rate (Cov), root mean squared error (RMSE). Coverage rate is calculated based on $\hat{\theta} \pm 1.96SE$.

	$\theta = 0$						$\theta = 0.2$					
Method	TIE	Mean	SD	SE	Cov	RMSE	Power	Mean	SD	SE	Cov	RMSE
$\rho = 0.2$ in the autoregressive LD structure												
cisMR-cML-BIC	0.110	0.000	0.043	0.027	0.890	0.043	0.994	0.206	0.048	0.029	0.888	0.049
**cisMR-cML**	0.042	0.001	0.042	0.046	0.958	0.042	0.934	0.204	0.043	0.049	0.970	0.043
cML-IND	0.388	-0.006	0.454	0.149	0.612	0.454	0.578	0.129	0.497	0.175	0.558	0.502
GEgger	0.074	-0.005	0.558	0.551	0.926	0.558	0.080	0.194	0.560	0.551	0.926	0.559
Egger-IND	0.192	-0.070	1.436	1.129	0.808	1.436	0.198	0.129	1.435	1.130	0.812	1.436
GIVW	0.102	0.003	0.337	0.312	0.898	0.337	0.138	0.200	0.337	0.312	0.896	0.337
IVW-IND	0.136	-0.026	0.579	0.515	0.864	0.579	0.158	0.170	0.582	0.516	0.858	0.582
LEgger	0.058	0.004	0.345	0.333	0.898	0.344	0.082	0.202	0.344	0.333	0.904	0.344
MR.LDP	0.000	0.001	0.064	NA	NA	0.064	0.000	0.031	0.060	NA	NA	0.179
MRAID	0.532	-0.018	0.573	NA	NA	0.573	0.762	0.148	0.544	NA	NA	0.546
MR.Corr2*	NA	0.000	0.000	0.000	0.000	0.000	NA	0.000	0.000	0.000	0.000	0.200
MR.CUE*	NA	0.000	0.000	0.000	0.000	0.000	NA	0.000	0.000	0.000	0.000	0.200
$\rho = 0.6$ in the autoregressive LD structure												
cisMR-cML-BIC	0.130	-0.001	0.068	0.031	0.870	0.068	0.982	0.205	0.069	0.034	0.868	0.069
**cisMR-cML**	0.030	0.002	0.053	0.061	0.970	0.053	0.864	0.205	0.056	0.064	0.972	0.056
GEgger	0.108	-0.003	0.479	0.445	0.892	0.478	0.128	0.194	0.478	0.445	0.898	0.477
GIVW	0.214	0.000	0.411	0.300	0.786	0.410	0.220	0.197	0.411	0.300	0.790	0.411
IVW-IND	0.932	0.098	1.303	0.045	0.068	1.305	0.944	0.295	1.302	0.045	0.072	1.304
LEgger	0.110	0.007	0.424	0.333	0.814	0.424	0.134	0.203	0.424	0.333	0.808	0.423
MR.LDP	0.000	0.001	0.069	NA	NA	0.069	0.000	0.035	0.074	NA	NA	0.181
MRAID	0.446	-0.024	0.545	NA	NA	0.545	0.658	0.071	0.550	NA	NA	0.564
MR.Corr2*	NA	0.000	0.000	0.000	0.000	0.000	NA	0.000	0.000	0.000	0.000	0.200
MR.CUE*	NA	0.000	0.000	0.000	0.000	0.000	NA	0.000	0.000	0.000	0.000	0.200
$\rho = 0.8$ in the autoregressive LD structure												
cisMR-cML-BIC	0.190	0.013	0.121	0.039	0.810	0.122	0.948	0.220	0.207	0.048	0.802	0.207
**cisMR-cML**	0.024	0.008	0.090	0.100	0.976	0.090	0.676	0.205	0.107	0.114	0.972	0.107
GEgger	0.182	0.018	0.546	0.399	0.818	0.546	0.202	0.213	0.546	0.399	0.820	0.545
GIVW	0.366	-0.001	0.500	0.282	0.634	0.500	0.364	0.196	0.501	0.282	0.634	0.501
IVW-IND	0.960	1.313	21.741	0.211	0.040	21.759	0.962	1.506	21.425	0.211	0.042	21.443
LEgger	0.190	0.014	0.540	0.339	0.750	0.539	0.196	0.208	0.539	0.339	0.756	0.538
MR.LDP	0.002	0.001	0.071	NA	NA	0.071	0.004	0.024	0.338	NA	NA	0.381
MRAID	0.266	-0.003	0.439	NA	NA	0.439	0.434	0.098	0.457	NA	NA	0.468
MR.Corr2*	NA	0.000	0.000	0.000	0.000	0.000	NA	0.000	0.000	0.000	0.000	0.200
MR.CUE*	NA	0.000	0.000	0.000	0.000	0.000	NA	0.000	0.000	0.000	0.000	0.200

*Traceplots for MR.Corr2 and MR.CUE gibbs sampler were flat at zero across all 500 simulation replicates in this setup.

Table S2. **Simulation results in scenario 1, where all 10 IVs have an effect on the exposure, and 4 invalid IVs ($K_1 = 4$).** Type-I error (T1E) or power, mean and standard deviation (SD) of estimates, mean standard error (SE) and coverage rate (Cov), root mean squared error (RMSE).

S3.2 Results for cisMR-cML with marginal genetic effects implementation

In this section, we investigated the performance of cisMR-cML-Marg if marginal GWAS summary statistics were modeled (Eq. (12) in the main text) in scenario 1 of the first set of simulation studies. Columns 'TP' and 'FP' in Tables S3 and S4 showed the average of correctly selected conditionally invalid IVs and incorrectly selected conditionally valid IVs

across 500 simulation replicates. When no invalid IV was present (Table S3), both cisMR-cML and cisMR-cML-Marg performed similarly well. This is attributed to the absence of additional pleiotropy introduced when employing marginal statistics with all IVs being valid. However, in the presence of 4 invalid IVs (Table S4), cisMR-cML-Marg had highly inflated type-I errors as the LD correlation increased. Despite only 4 out of the 10 SNPs being invalid, the plurality assumption was violated in cisMR-cML-Marg with the use of marginal statistics. This occurred because, when the pleiotropic effects of the invalid IVs were absorbed in the marginal genetic effects, all IVs became invalid and the model imposed by cisMR-cML-Marg was not identifiable (see Section "Modeling conditional effects versus marginal effects" in the main text). As shown in the 'FP' column, cisMR-cML-Marg-BIC selected many conditionally valid IVs (which were marginally invalid IVs).

	$\theta = 0$								$\theta = 0.2$							
Method	TIE	Mean	SD	SE	Cov	RMSE	TP	FP	Power	Mean	SD	SE	Cov	RMSE	TP	FP
$\rho = 0.2$ in the autoregressive LD structure																
cisMR-cML-BIC	0.056	0.000	0.020	0.020	0.944	0.020	0.000	0.024	1.000	0.200	0.022	0.021	0.948	0.022	0.000	0.016
cisMR-cML	0.020	-0.001	0.020	0.026	0.980	0.020			0.998	0.200	0.022	0.028	0.978	0.022		
cisMR-cML-Marg-BIC	0.056	0.000	0.020	0.020	0.944	0.020	0.000	0.022	1.000	0.200	0.022	0.021	0.948	0.022	0.000	0.014
cisMR-cML-Marg	0.020	0.000	0.020	0.025	0.980	0.020			1.000	0.200	0.022	0.026	0.976	0.022		
$\rho = 0.6$ in the autoregressive LD structure																
cisMR-cML-BIC	0.052	0.000	0.020	0.020	0.948	0.020	0.000	0.028	1.000	0.200	0.022	0.021	0.952	0.022	0.000	0.018
cisMR-cML	0.012	-0.001	0.020	0.030	0.988	0.020			0.998	0.200	0.023	0.032	0.984	0.023		
cisMR-cML-Marg-BIC	0.048	-0.001	0.019	0.020	0.952	0.019	0.000	0.012	1.000	0.200	0.021	0.021	0.954	0.021	0.000	0.008
cisMR-cML-Marg	0.030	0.000	0.019	0.023	0.970	0.019			1.000	0.201	0.022	0.025	0.966	0.022		
$\rho = 0.8$ in the autoregressive LD structure																
cisMR-cML-BIC	0.038	0.000	0.020	0.020	0.962	0.020	0.000	0.012	1.000	0.200	0.023	0.021	0.954	0.022	0.000	0.012
cisMR-cML	0.006	-0.001	0.022	0.037	0.994	0.022			0.994	0.200	0.024	0.038	0.994	0.024		
cisMR-cML-Marg-BIC	0.034	-0.001	0.019	0.020	0.966	0.019	0.000	0.014	1.000	0.200	0.021	0.021	0.958	0.021	0.000	0.008
cisMR-cML-Marg	0.022	-0.001	0.019	0.021	0.978	0.019			1.000	0.200	0.022	0.024	0.974	0.022		

Table S3. **Comparison between cisMR-cML and cisMR-cML-Marg in scenario 1, where all 10 IVs have an effect on the exposure, and no invalid IV ($K_1 = 0$).** Type-I error (T1E) or power, mean and standard deviation (SD) of estimates, mean standard error (SE) and coverage rate (Cov), root mean squared error (RMSE), mean of true positives (TP) and false positives (FP) across 500 replicates.

Method	$\theta = 0$								$\theta = 0.2$							
	TIE	Mean	SD	SE	Cov	RMSE	TP	FP	Power	Mean	SD	SE	Cov	RMSE	TP	FP
$\rho = 0.2$ in the autoregressive LD structure																
cisMR-cML-BIC	0.110	0.000	0.043	0.027	0.890	0.043	3.644	0.018	0.994	0.206	0.048	0.029	0.888	0.049	3.612	0.038
cisMR-cML	0.042	0.001	0.042	0.046	0.958	0.042			0.934	0.204	0.043	0.049	0.970	0.043		
cisMR-cML-Marg-BIC	0.624	-0.006	0.210	0.034	0.376	0.210	3.518	2.152	0.898	0.195	0.209	0.036	0.368	0.209	3.486	2.036
cisMR-cML-Marg	0.190	-0.004	0.166	0.122	0.810	0.165			0.506	0.184	0.165	0.123	0.790	0.166		
$\rho = 0.6$ in the autoregressive LD structure																
cisMR-cML-BIC	0.130	-0.001	0.068	0.031	0.870	0.068	3.560	0.050	0.982	0.205	0.069	0.034	0.868	0.069	3.522	0.052
cisMR-cML	0.030	0.002	0.053	0.061	0.970	0.053			0.864	0.205	0.056	0.064	0.972	0.056		
cisMR-cML-Marg-BIC	0.903	-0.002	0.477	0.042	0.097	0.477	3.170	3.536	0.923	0.124	0.488	0.043	0.091	0.493	3.146	3.534
cisMR-cML-Marg	0.554	-0.001	0.415	0.164	0.446	0.414			0.634	0.126	0.440	0.165	0.418	0.446		
$\rho = 0.8$ in the autoregressive LD structure																
cisMR-cML-BIC	0.190	0.013	0.121	0.039	0.810	0.122	3.380	0.056	0.948	0.220	0.207	0.048	0.802	0.207	3.262	0.080
cisMR-cML	0.024	0.008	0.090	0.100	0.976	0.090			0.676	0.205	0.107	0.114	0.972	0.107		
cisMR-cML-Marg-BIC	0.959	-0.009	0.627	0.044	0.041	0.627	2.742	3.382	0.964	0.182	0.721	0.049	0.043	0.721	2.672	3.246
cisMR-cML-Marg	0.783	0.000	0.655	0.172	0.217	0.654			0.776	0.197	0.769	0.168	0.202	0.768		

Table S4. **Comparison between cisMR-cML and cisMR-cML-Marg in scenario 1, where all 10 IVs have an effect on the exposure, and 4 invalid IVs ($K_1 = 4$).** Type-I error (T1E) or power, mean and standard deviation (SD) of estimates, mean standard error (SE) and coverage rate (Cov), root mean squared error (RMSE), mean of true positives (TP) and false positives (FP) across 500 replicates.

S3.3 Results for Lasso implementation

In this section, we investigated the performance of cisMR-Lasso described in Section S2 in scenario 1 of the first set of simulation studies. Particularly, we compared the performance of invalid IV selection for cisMR-cML and cisMR-Lasso using BIC criteria respectively by calculating the number of correctly selected invalid IVs (true positives) and the number of incorrectly selected invalid IVs (false positives). Columns 'TP' and 'FP' in Tables S5 and S6 showed the average of true positives and false positives across 500 simulation replicates. When no invalid IV was present (Table S5), both cisMR-cML and cisMR-Lasso performed similarly well. However, in the presence of 4 invalid IVs (Table S6), cisMR-Lasso had inflated type-I errors and biased estimates. Furthermore, cisMR-cML-BIC outperformed cisMR-Lasso-BIC in the selection of invalid IVs, suggested by a higher TP and a lower FP.

	$\theta = 0$								$\theta = 0.2$							
Method	TIE	Mean	SD	SE	Cov	RMSE	TP	FP	Power	Mean	SD	SE	Cov	RMSE	TP	FP
$\rho = 0.2$ in the autoregressive LD structure																
cisMR-cML-BIC	0.056	0.000	0.020	0.020	0.944	0.020	0.000	0.024	1.000	0.200	0.022	0.021	0.948	0.022	0.000	0.016
cisMR-cML	0.020	-0.001	0.020	0.026	0.980	0.020			0.998	0.200	0.022	0.028	0.978	0.022		
cisMR-Lasso-BIC	0.056	0.000	0.020	0.020	0.944	0.020	0.000	0.024	1.000	0.200	0.022	0.021	0.948	0.022	0.000	0.010
cisMR-Lasso	0.040	-0.001	0.020	0.023	0.960	0.020			1.000	0.200	0.022	0.025	0.972	0.022		
$\rho = 0.6$ in the autoregressive LD structure																
cisMR-cML-BIC	0.052	0.000	0.020	0.020	0.948	0.020	0.000	0.028	1.000	0.200	0.022	0.021	0.952	0.022	0.000	0.018
cisMR-cML	0.012	-0.001	0.020	0.030	0.988	0.020			0.998	0.200	0.023	0.032	0.984	0.023		
cisMR-Lasso-BIC	0.050	0.000	0.019	0.020	0.950	0.019	0.000	0.024	1.000	0.200	0.022	0.021	0.952	0.022	0.000	0.010
cisMR-Lasso	0.020	-0.001	0.020	0.025	0.980	0.020			1.000	0.200	0.022	0.025	0.964	0.022		
$\rho = 0.8$ in the autoregressive LD structure																
cisMR-cML-BIC	0.038	0.000	0.020	0.020	0.962	0.020	0.000	0.012	1.000	0.200	0.023	0.021	0.954	0.022	0.000	0.012
cisMR-cML	0.006	-0.001	0.022	0.037	0.994	0.022			0.994	0.200	0.024	0.038	0.994	0.024		
cisMR-Lasso-BIC	0.038	0.000	0.020	0.020	0.962	0.020	0.000	0.006	1.000	0.200	0.021	0.021	0.958	0.021	0.000	0.000
cisMR-Lasso	0.020	0.000	0.020	0.026	0.980	0.020			1.000	0.200	0.022	0.028	0.982	0.022		

Table S5. **Comparison between cisMR-cML and cisMR-Lasso in scenario 1, where all 10 IVs have an effect on the exposure, and no invalid IV ($K_1 = 0$).** Type-I error (T1E) or power, mean and standard deviation (SD) of estimates, mean standard error (SE) and coverage rate (Cov), root mean squared error (RMSE), mean of true positives (TP) and false positives (FP) across 500 replicates.

	$\theta = 0$								$\theta = 0.2$							
Method	TIE	Mean	SD	SE	Cov	RMSE	TP	FP	Power	Mean	SD	SE	Cov	RMSE	TP	FP
$\rho = 0.2$ in the autoregressive LD structure																
cisMR-cML-BIC	0.110	0.000	0.043	0.027	0.890	0.043	3.644	0.018	0.994	0.206	0.048	0.029	0.888	0.049	3.612	0.038
cisMR-cML	0.042	0.001	0.042	0.046	0.958	0.042			0.934	0.204	0.043	0.049	0.970	0.043		
cisMR-Lasso-BIC	0.203	0.000	0.246	0.031	0.797	0.246	3.482	0.496	0.984	0.194	0.588	0.058	0.808	0.587	3.484	0.554
cisMR-Lasso	0.098	-0.001	0.208	0.079	0.902	0.208			0.866	0.228	0.195	0.079	0.908	0.197		
$\rho = 0.6$ in the autoregressive LD structure																
cisMR-cML-BIC	0.130	-0.001	0.068	0.031	0.870	0.068	3.560	0.050	0.982	0.205	0.069	0.034	0.868	0.069	3.522	0.052
cisMR-cML	0.030	0.002	0.053	0.061	0.970	0.053			0.864	0.205	0.056	0.064	0.972	0.056		
cisMR-Lasso-BIC	0.260	-0.003	0.286	0.038	0.740	0.286	3.318	0.778	0.968	0.231	0.261	0.041	0.761	0.262	3.366	0.892
cisMR-Lasso	0.108	0.004	0.269	0.125	0.892	0.269			0.758	0.248	0.275	0.128	0.876	0.279		
$\rho = 0.8$ in the autoregressive LD structure																
cisMR-cML-BIC	0.190	0.013	0.121	0.039	0.810	0.122	3.380	0.056	0.948	0.220	0.207	0.048	0.802	0.207	3.262	0.080
cisMR-cML	0.024	0.008	0.090	0.100	0.976	0.090			0.676	0.205	0.107	0.114	0.972	0.107		
cisMR-Lasso-BIC	0.376	0.000	0.999	0.084	0.624	0.997	2.958	1.112	0.949	0.347	1.001	0.088	0.591	1.011	2.806	1.126
cisMR-Lasso	0.134	0.027	0.572	0.366	0.866	0.572			0.574	0.304	0.670	0.384	0.846	0.678		

Table S6. **Comparison between cisMR-cML and cisMR-Lasso in scenario 1, where all 10 IVs have an effect on the exposure, and 4 invalid IVs ($K_1 = 4$).** Type-I error (T1E) or power, mean and standard deviation (SD) of estimates, mean standard error (SE) and coverage rate (Cov), root mean squared error (RMSE), mean of true positives (TP) and false positives (FP) across 500 replicates.

S3.4 Full results for scenario 2

Method	$\theta = 0$								$\theta = 0.2$							
	TIE	Mean	SD	SE	Cov	RMSE	TP	FP	Power	Mean	SD	SE	Cov	RMSE	TP	FP
$\rho = 0.2$ in the autoregressive LD structure																
cisMR-cML-BIC	0.040	0.002	0.020	0.020	0.960	0.020	1.920	0.020	1.000	0.201	0.022	0.022	0.956	0.022	1.904	0.016
cisMR-cML	0.022	0.002	0.021	0.027	0.978	0.021			1.000	0.202	0.023	0.029	0.988	0.023		
cisMR-cML-X	0.096	0.008	0.088	0.069	0.904	0.089			0.794	0.206	0.084	0.071	0.890	0.084		
GEgger	0.000	0.002	0.272	0.426	1.000	0.272			0.012	0.204	0.273	0.426	1.000	0.272		
GEgger-X	0.116	-0.009	0.190	0.167	0.884	0.190			0.348	0.190	0.192	0.168	0.878	0.192		
GIVW	0.000	0.001	0.107	0.329	1.000	0.106			0.000	0.198	0.106	0.329	1.000	0.106		
GIVW-X	0.118	0.000	0.092	0.084	0.882	0.092			0.590	0.199	0.092	0.084	0.886	0.092		
LEgger	0.000	-0.012	0.173	0.348	1.000	0.173			0.002	0.185	0.173	0.348	1.000	0.174		
LEgger-X	0.064	-0.005	0.104	0.093	0.836	0.104			0.298	0.193	0.104	0.094	0.846	0.104		
$\rho = 0.6$ in the autoregressive LD structure																
cisMR-cML-BIC	0.068	0.002	0.024	0.022	0.932	0.024	1.914	0.028	1.000	0.202	0.026	0.024	0.920	0.026	1.894	0.024
cisMR-cML	0.016	0.001	0.024	0.033	0.984	0.024			0.994	0.202	0.026	0.036	0.976	0.026		
cisMR-cML-X	0.118	0.008	0.155	0.108	0.882	0.155			0.680	0.188	0.157	0.109	0.870	0.157		
GEgger	0.022	-0.032	0.324	0.415	0.978	0.325			0.036	0.165	0.324	0.415	0.980	0.326		
GEgger-X	0.138	-0.026	0.523	0.436	0.862	0.523			0.198	0.172	0.525	0.436	0.868	0.525		
GIVW	0.062	0.013	0.303	0.314	0.938	0.303			0.122	0.210	0.302	0.314	0.940	0.302		
GIVW-X	0.192	0.013	0.299	0.240	0.808	0.299			0.270	0.212	0.298	0.241	0.802	0.298		
LEgger	0.014	-0.014	0.329	0.340	0.948	0.329			0.052	0.182	0.329	0.340	0.944	0.329		
LEgger-X	0.074	-0.015	0.336	0.277	0.802	0.336			0.120	0.183	0.337	0.278	0.802	0.337		
$\rho = 0.8$ in the autoregressive LD structure																
cisMR-cML-BIC	0.082	0.002	0.031	0.026	0.918	0.031	1.873	0.040	0.996	0.202	0.036	0.028	0.912	0.036	1.868	0.032
cisMR-cML	0.014	0.002	0.031	0.043	0.986	0.031			0.966	0.202	0.034	0.046	0.976	0.034		
cisMR-cML-X	0.138	0.004	0.202	0.115	0.862	0.201			0.633	0.185	0.208	0.121	0.846	0.208		
GEgger	0.120	-0.015	0.440	0.386	0.880	0.440			0.142	0.179	0.439	0.386	0.882	0.439		
GEgger-X	0.202	-0.015	0.595	0.491	0.798	0.595			0.200	0.181	0.594	0.492	0.810	0.593		
GIVW	0.288	0.031	0.455	0.291	0.712	0.455			0.338	0.227	0.454	0.291	0.710	0.454		
GIVW-X	0.310	0.031	0.456	0.297	0.690	0.457			0.340	0.229	0.455	0.298	0.684	0.456		
LEgger	0.094	-0.009	0.453	0.339	0.836	0.453			0.128	0.184	0.453	0.339	0.834	0.453		
LEgger-X	0.102	-0.021	0.518	0.372	0.748	0.518			0.140	0.174	0.520	0.372	0.756	0.520		

Table S7. **Simulation results in scenario 2 with $|\mathcal{I}_X| = 5, |\mathcal{I}_Y \setminus \mathcal{I}_X| = 2$ and $|\mathcal{I}_X \cap \mathcal{I}_Y| = 0$.** Type-I error (T1E) or power, mean and standard deviation (SD) of estimates, mean standard error (SE) and coverage rate (Cov), root mean squared error (RMSE), mean of true positives (TP) and false positives (FP) across 500 replicates.

	$\theta = 0$								$\theta = 0.2$							
Method	TIE	Mean	SD	SE	Cov	RMSE	TP	FP	Power	Mean	SD	SE	Cov	RMSE	TP	FP
$\rho = 0.2$ in the autoregressive LD structure																
cisMR-cML-BIC	0.070	0.000	0.028	0.023	0.930	0.028	2.802	0.026	0.998	0.202	0.031	0.025	0.930	0.031	2.780	0.018
cisMR-cML	0.026	0.000	0.029	0.038	0.974	0.029			0.950	0.200	0.032	0.041	0.984	0.032		
cisMR-cML-X	0.112	0.011	0.095	0.073	0.888	0.096			0.758	0.203	0.089	0.077	0.914	0.089		
GEgger	0.056	0.028	0.369	0.419	0.944	0.370			0.096	0.230	0.369	0.419	0.946	0.370		
GEgger-X	0.146	0.056	0.565	0.459	0.854	0.567			0.202	0.255	0.566	0.460	0.860	0.568		
GIVW	0.064	0.030	0.267	0.318	0.936	0.268			0.098	0.227	0.268	0.318	0.938	0.269		
GIVW-X	0.138	0.033	0.267	0.236	0.862	0.269			0.302	0.231	0.268	0.237	0.858	0.269		
LEgger	0.042	0.017	0.287	0.336	0.928	0.287			0.062	0.214	0.288	0.336	0.928	0.288		
LEgger-X	0.044	0.014	0.340	0.276	0.868	0.340			0.100	0.212	0.340	0.276	0.862	0.340		
$\rho = 0.6$ in the autoregressive LD structure																
cisMR-cML-BIC	0.080	0.005	0.064	0.026	0.920	0.064	2.762	0.020	0.996	0.203	0.042	0.029	0.916	0.042	2.732	0.032
cisMR-cML	0.010	0.001	0.039	0.048	0.990	0.039			0.926	0.200	0.044	0.056	0.980	0.044		
cisMR-cML-X	0.172	0.016	0.173	0.115	0.828	0.173			0.634	0.196	0.178	0.113	0.830	0.178		
GEgger	0.086	0.008	0.397	0.408	0.914	0.397			0.116	0.204	0.397	0.409	0.916	0.397		
GEgger-X	0.138	0.043	0.607	0.554	0.862	0.608			0.164	0.241	0.607	0.555	0.864	0.608		
GIVW	0.180	0.048	0.370	0.305	0.820	0.373			0.196	0.244	0.371	0.305	0.820	0.373		
GIVW-X	0.190	0.049	0.372	0.303	0.810	0.375			0.254	0.247	0.373	0.304	0.814	0.376		
LEgger	0.078	0.022	0.372	0.335	0.880	0.372			0.100	0.218	0.371	0.335	0.878	0.372		
LEgger-X	0.046	0.014	0.442	0.363	0.830	0.442			0.068	0.211	0.444	0.363	0.832	0.444		
$\rho = 0.8$ in the autoregressive LD structure																
cisMR-cML-BIC	0.116	0.006	0.124	0.032	0.884	0.124	2.662	0.038	0.980	0.204	0.099	0.039	0.872	0.099	2.646	0.056
cisMR-cML	0.010	0.003	0.066	0.070	0.990	0.066			0.826	0.200	0.082	0.082	0.982	0.082		
cisMR-cML-X	0.180	0.023	0.243	0.145	0.820	0.244			0.586	0.199	0.254	0.142	0.804	0.254		
GEgger	0.164	0.024	0.494	0.383	0.836	0.494			0.182	0.218	0.494	0.383	0.838	0.494		
GEgger-X	0.172	0.026	0.677	0.557	0.828	0.677			0.196	0.223	0.679	0.558	0.830	0.679		
GIVW	0.352	0.060	0.487	0.284	0.648	0.490			0.366	0.256	0.488	0.285	0.644	0.491		
GIVW-X	0.306	0.061	0.491	0.327	0.694	0.495			0.320	0.259	0.492	0.328	0.702	0.495		
LEgger	0.140	0.024	0.476	0.340	0.806	0.476			0.168	0.218	0.475	0.341	0.808	0.475		
LEgger-X	0.058	0.018	0.575	0.429	0.808	0.575			0.094	0.214	0.576	0.429	0.800	0.575		

Table S8. **Simulation results in scenario 2 with $|\mathcal{I}_X| = 5, |\mathcal{I}_Y \setminus \mathcal{I}_X| = 2$ and $|\mathcal{I}_X \cap \mathcal{I}_Y| = 1$.** Type-I error (T1E) or power, mean and standard deviation (SD) of estimates, mean standard error (SE) and coverage rate (Cov), root mean squared error (RMSE), mean of true positives (TP) and false positives (FP) across 500 replicates.

S3.5 Different sets of SNPs used in cisMR-cML in scenario 2

Recall that in the second scenario described in the simulation set-up in the main text, we simulated 10 SNPs, $|\mathcal{I}_X| = 5$ SNPs were associated with the exposure, and $K_2 = |\mathcal{I}_Y \setminus \mathcal{I}_X| = 2$ SNPs were only associated with the outcome, and 3 were not associated with either X or Y. We considered three ways to select the set of SNPs to be used in the analysis: (1) using all 10 SNPs, i.e. the implementation in the main text; (2) using the 7 SNPs in $\mathcal{I}_X \cup \mathcal{I}_Y$; (3) using SNPs in $\mathcal{I}_{X_s} \cup \mathcal{I}_Y$, where $\mathcal{I}_{X_s}$ is a subset of $\mathcal{I}_X$ and $|\mathcal{I}_{X_s}| = 3$. We calculated the joint effect estimates $(\hat{\boldsymbol{\beta}}_X, \boldsymbol{\Sigma}_X, \hat{\boldsymbol{\beta}}_Y, \boldsymbol{\Sigma}_Y)$ based on the above three different sets of SNPs. Then we applied cisMR-cML with these three sets of estimates and referred to them as cisMR-cML1, cisMR-cML2, and cisMR-cML3, respectively. As in the main simulation, we implemented cisMR-cML with 5 random starts and $B = 100$ data perturbations.

As shown in Table S9, cisMR-cML based on all three different sets of SNPs yielded well-controlled type-I errors and (almost) unbiased estimates. This suggested that as long as we correctly included SNPs in $\mathcal{I}_Y$ in the analysis, cisMR-cML will be robust even though we included some irrelevant/null SNPs (in cisMR-cML1) or omitted some valid (and relevant) SNPs (in cisMR-cML3). However, we can see that cisMR-cML2 which was based on the second set of SNPs (i.e. $\mathcal{I}_X \cup \mathcal{I}_Y$) was the most powerful, followed by cisMR-cML1 and cisMR-cML3. This suggested that using irrelevant SNPs may induce more variation in the proposed method, and omitting some true (conditionally) relevant and valid SNPs will also lose power.

(a) $K_1 = 0$

ρ	θ	cisMR-cML1	cisMR-cML2	cisMR-cML3
0.2	0.00	0.022<br>0.002<br>0.021	0.020<br>0.002<br>0.021	0.028<br>0.002<br>0.036
	0.05	0.490<br>0.051<br>0.021	0.544<br>0.052<br>0.021	0.344<br>0.052<br>0.037
	0.10	0.956<br>0.102<br>0.021	0.956<br>0.102<br>0.021	0.798<br>0.102<br>0.037
0.6	0.00	0.016<br>0.001<br>0.024	0.024<br>0.002<br>0.024	0.020<br>0.001<br>0.048
	0.05	0.364<br>0.052<br>0.024	0.408<br>0.052<br>0.024	0.272<br>0.052<br>0.053
	0.10	0.866<br>0.102<br>0.025	0.900<br>0.102<br>0.025	0.730<br>0.102<br>0.052

(b) $K_1 = 1$

ρ	θ	cisMR-cML1	cisMR-cML2	cisMR-cML3
0.2	0.00	0.026<br>0.000<br>0.029	0.018<br>0.001<br>0.030	0.046<br>0.002<br>0.109
	0.05	0.354<br>0.050<br>0.030	0.406<br>0.050<br>0.030	0.290<br>0.052<br>0.115
	0.10	0.814<br>0.100<br>0.030	0.852<br>0.101<br>0.030	0.652<br>0.100<br>0.122
0.6	0.00	0.010<br>0.001<br>0.039	0.028<br>0.002<br>0.043	0.052<br>0.006<br>0.199
	0.05	0.276<br>0.051<br>0.039	0.320<br>0.052<br>0.047	0.242<br>0.054<br>0.193
	0.10	0.694<br>0.100<br>0.039	0.746<br>0.101<br>0.042	0.570<br>0.100<br>0.213

Table S9. Simulation results of different implementations of cisMR-cML in scenario 2 with $|\mathcal{I}_X| = 5, |\mathcal{I}_Y \setminus \mathcal{I}_X| = 2$. Left: $|\mathcal{I}_X \cap \mathcal{I}_Y| = 0$. Right: $|\mathcal{I}_X \cap \mathcal{I}_Y| = 1$. In each cell, from top to bottom are empirical type-I error/power, mean($\hat{\theta}$), RMSE.

S3.6 Full results for the three additional scenarios

	$\theta = 0$						$\theta = 0.2$					
Method	TIE	Mean	SD	SE	Cov	RMSE	Power	Mean	SD	SE	Cov	RMSE
**$N_X = 5000$, average F-statistics across 500 replicates is 27.30**												
cisMR-cML-BIC	0.056	0.000	0.021	0.020	0.944	0.021	1.000	0.201	0.024	0.023	0.952	0.024
cisMR-cML	0.012	-0.001	0.021	0.033	0.988	0.021	0.994	0.201	0.025	0.037	0.986	0.025
GEgger	0.038	-0.001	0.030	0.031	0.962	0.030	0.994	0.192	0.035	0.034	0.926	0.036
GIVW	0.038	0.000	0.018	0.021	0.962	0.018	1.000	0.194	0.023	0.023	0.928	0.023
LEgger	0.052	0.000	0.025	0.023	0.902	0.025	0.994	0.192	0.029	0.027	0.894	0.030
MR.LDP	0.028	-0.001	0.019	NA	NA	0.019	1.000	0.200	0.024	NA	NA	0.024
MRAID	0.028	-0.001	0.025	NA	NA	0.025	0.998	0.199	0.024	NA	NA	0.024
MR.Corr2*	0.042	-0.001	0.019	0.020	0.958	0.019	0.995	0.078	0.092	0.010	0.400	0.153
MR.CUE	0.024	0.001	0.019	0.024	0.976	0.019	1.000	0.204	0.024	0.029	0.978	0.025
**$N_X = 1000$, average F-statistics across 500 replicates is 6.25**												
cisMR-cML-BIC	0.056	0.000	0.024	0.021	0.944	0.024	1.000	0.204	0.039	0.036	0.922	0.039
cisMR-cML	0.002	-0.001	0.025	0.051	0.998	0.025	0.942	0.201	0.039	0.060	0.988	0.039
GEgger	0.040	0.000	0.028	0.029	0.960	0.028	0.950	0.169	0.047	0.043	0.806	0.057
GIVW	0.026	0.000	0.017	0.020	0.974	0.017	1.000	0.173	0.029	0.030	0.802	0.040
LEgger	0.050	-0.001	0.023	0.022	0.906	0.023	0.958	0.164	0.038	0.035	0.772	0.052
MR.LDP	0.010	0.000	0.021	NA	NA	0.020	0.974	0.200	0.040	NA	NA	0.040
MRAID	0.004	0.001	0.024	NA	NA	0.024	0.694	0.158	0.062	NA	NA	0.075
MR.Corr2*	0.020	-0.001	0.022	0.024	0.980	0.022	0.994	0.060	0.090	0.012	0.284	0.167
MR.CUE	0.002	1.29E+55	2.87E+56	1.41E+56	0.998	2.87E+56	0.998	0.217	0.046	0.050	0.974	0.049
**$N_X = 500$, average F-statistics across 500 replicates is 3.62**												
cisMR-cML-BIC	0.048	0.000	0.031	0.022	0.952	0.031	1.000	0.208	0.055	0.049	0.908	0.055
cisMR-cML	0.000	-0.002	0.026	0.066	1.000	0.026	0.768	0.198	0.049	0.085	0.978	0.049
GEgger	0.044	0.000	0.027	0.028	0.956	0.027	0.856	0.146	0.052	0.049	0.712	0.075
GIVW	0.014	0.000	0.016	0.019	0.986	0.016	0.998	0.152	0.032	0.034	0.632	0.058
LEgger	0.042	-0.001	0.022	0.020	0.910	0.022	0.862	0.139	0.044	0.039	0.588	0.075
MR.LDP	0.004	0.000	0.023	NA	NA	0.023	0.632	0.130	0.074	NA	NA	0.102
MRAID	0.000	0.000	0.015	NA	NA	0.015	0.436	0.117	0.064	NA	NA	0.105
MR.Corr2*	0.004	0.000	0.028	0.035	0.996	0.028	1.000	0.040	0.081	0.011	0.182	0.179
MR.CUE	0.002	5.44E+61	1.19E+63	7.17E+62	0.998	1.19E+63	0.800	2.99E+37	6.61E+38	2.47E+38	0.966	6.61E+38

*Among 500 simulation replicates, the traceplots for MR.Corr2 gibbs sampler were flat at zero in 289, 342 and 396 replicates when $N_X = 5000, 1000, 500$ respectively and $\theta = 0.2$.

Table S10. **Simulation results for weak-IV scenario with reduced exposure GWAS sample size N_X, under the setup of $\rho = 0.6$ in the autoregressive LD structure and no invalid IV.** Type-I error (T1E) or power, mean and standard deviation (SD) of estimates, mean standard error (SE) and coverage rate (Cov), root mean squared error (RMSE).

	$\theta = 0$						$\theta = 0.2$					
Method	TIE	Mean	SD	SE	Cov	RMSE	Power	Mean	SD	SE	Cov	RMSE
$h_X^2 = 0.01$, **average F-statistics across 500 replicates is 11.09**												
cisMR-cML-BIC	0.052	0.000	0.048	0.045	0.948	0.048	0.998	0.203	0.051	0.049	0.956	0.051
cisMR-cML	0.006	-0.001	0.049	0.078	0.994	0.049	0.784	0.201	0.054	0.080	0.984	0.054
GEgger	0.042	-0.001	0.065	0.067	0.958	0.065	0.746	0.182	0.071	0.071	0.932	0.073
GIVW	0.036	-0.001	0.040	0.046	0.964	0.040	0.990	0.185	0.045	0.048	0.956	0.048
LEgger	0.056	-0.001	0.054	0.050	0.904	0.054	0.822	0.179	0.058	0.055	0.904	0.062
MR.LDP	0.018	-0.001	0.044	NA	NA	0.044	0.938	0.200	0.051	NA	NA	0.051
MRAID	0.006	0.010	0.092	NA	NA	0.093	0.680	0.181	0.119	NA	NA	0.120
MR.Corr2	0.032	-0.001	0.044	0.047	0.968	0.044	0.994	0.203	0.051	0.052	0.960	0.051
MR.CUE	0.010	0.003	0.045	0.058	0.990	0.045	0.976	0.211	0.053	0.064	0.984	0.054
$h_X^2 = 0.005$, **average F-statistics across 500 replicates is 6.01**												
cisMR-cML-BIC	0.048	-0.002	0.071	0.064	0.952	0.071	0.884	0.206	0.078	0.071	0.960	0.078
cisMR-cML	0.002	-0.002	0.074	0.125	0.998	0.074	0.336	0.202	0.082	0.128	0.994	0.082
GEgger	0.042	-0.001	0.090	0.092	0.958	0.090	0.448	0.167	0.099	0.097	0.924	0.104
GIVW	0.024	-0.001	0.055	0.063	0.976	0.055	0.766	0.171	0.061	0.066	0.940	0.067
LEgger	0.050	-0.002	0.073	0.068	0.906	0.073	0.494	0.161	0.079	0.074	0.892	0.087
MR.LDP	0.008	-0.001	0.065	NA	NA	0.065	0.690	0.201	0.076	NA	NA	0.076
MRAID	0.002	0.020	0.195	NA	NA	0.196	0.290	0.212	0.236	NA	NA	0.236
MR.Corr2	0.024	-0.002	0.065	0.070	0.976	0.065	0.844	0.203	0.072	0.076	0.972	0.072
MR.CUE	0.002	1.10E+56	2.46E+57	1.20E+57	0.998	2.46E+57	0.652	4.15E+56	9.28E+57	5.50E+57	0.990	9.28E+57

*Among 500 simulation replicates, the traceplots for MR.Corr2 gibbs sampler were flat at zero in 289, 342 and 396 replicates when $N_X = 5000, 1000, 500$ respectively and $\theta = 0.2$.

Table S11. **Simulation results for weak-IV scenario with reduced IV-exposure effect size (determined by h_X^2), under the setup of $\rho = 0.6$ in the autoregressive LD structure and no invalid IV.** Type-I error (T1E) or power, mean and standard deviation (SD) of estimates, mean standard error (SE) and coverage rate (Cov), root mean squared error (RMSE).

Method	θ = 0								θ = 0.2							
	TIE	Mean	SD	SE	Cov	RMSE	TP	FP	Power	Mean	SD	SE	Cov	RMSE	TP	FP
								$N_Y = 5e4$								
cisMR-cML-BIC	0.248	0.001	0.034	0.020	0.752	0.034	0.208	0.068	1.000	0.204	0.037	0.022	0.748	0.038	0.17	0.058
cisMR-cML	0.040	0.002	0.031	0.036	0.960	0.031			0.994	0.204	0.032	0.038	0.968	0.032		
GEgger	0.090	0.004	0.038	0.036	0.910	0.038			0.996	0.199	0.039	0.038	0.928	0.039		
GIVW	0.144	0.001	0.031	0.025	0.856	0.031			1.000	0.198	0.033	0.026	0.848	0.033		
LEgger	0.068	0.001	0.029	0.025	0.882	0.029			0.994	0.196	0.031	0.027	0.874	0.031		
MRLDP	0.072	0.001	0.030	NA	NA	0.030			1.000	0.201	0.032	NA	NA	0.032		
MRAID	0.154	0.001	0.034	NA	NA	0.034			0.998	0.203	0.037	NA	NA	0.037		
MRCUE	0.066	0.002	0.029	0.029	0.934	0.029			1.000	0.204	0.031	0.031	0.938	0.031		
								$N_Y = 1e5$								
cisMR-cML-BIC	0.248	0.001	0.024	0.014	0.752	0.024	0.208	0.068	1.000	0.203	0.027	0.017	0.772	0.027	0.126	0.046
cisMR-cML	0.042	0.001	0.022	0.026	0.958	0.022			1.000	0.204	0.024	0.028	0.964	0.024		
GEgger	0.090	0.003	0.027	0.025	0.910	0.027			1.000	0.198	0.029	0.028	0.922	0.029		
GIVW	0.144	0.001	0.022	0.017	0.856	0.022			1.000	0.198	0.024	0.019	0.848	0.024		
LEgger	0.068	0.000	0.021	0.018	0.882	0.021			1.000	0.196	0.023	0.020	0.894	0.023		
MRLDP	0.072	0.001	0.021	NA	NA	0.021			1.000	0.201	0.024	NA	NA	0.024		
MRAID	0.134	0.002	0.026	NA	NA	0.026			0.998	0.202	0.029	NA	NA	0.029		
MRCUE	0.060	0.002	0.021	0.021	0.940	0.021			1.000	0.203	0.023	0.023	0.940	0.023		
								$N_Y = 5e5$								
cisMR-cML-BIC	0.242	0.000	0.011	0.006	0.758	0.011	0.204	0.064	1.000	0.202	0.015	0.011	0.870	0.015	0.044	0.028
cisMR-cML	0.038	0.001	0.010	0.012	0.962	0.010			1.000	0.202	0.014	0.016	0.964	0.014		
GEgger	0.090	0.001	0.012	0.011	0.910	0.012			1.000	0.197	0.017	0.017	0.922	0.017		
GIVW	0.144	0.000	0.010	0.008	0.856	0.010			1.000	0.197	0.013	0.011	0.864	0.014		
LEgger	0.068	0.000	0.009	0.008	0.882	0.009			1.000	0.196	0.014	0.013	0.896	0.015		
MRLDP	0.072	0.000	0.009	NA	NA	0.009			1.000	0.201	0.014	NA	NA	0.014		
MRAID	0.128	0.000	0.014	NA	NA	0.014			1.000	0.201	0.018	NA	NA	0.018		
MRCUE	0.038	0.001	0.009	0.010	0.962	0.009			1.000	0.202	0.013	0.014	0.962	0.013		

*Among 500 simulation replicates, MR.Corr2 often failed to converge thus results are not shown.

Table S12. **Simulation results for weak invalid IV scenario with $r_i = \kappa/\sqrt{N_Y}$, $\kappa = 1$ under the setup of $\rho = 0.6$ in the autoregressive LD structure and 4 invalid IVs, $N_X = 1e4$ samples.** Type-I error (T1E) or power, mean and standard deviation (SD) of estimates, mean standard error (SE) and coverage rate (Cov), root mean squared error (RMSE), mean of true positives (TP) and false positives (FP).

Method	θ = 0 TIE	Mean	SD	SE	Cov	RMSE	TP	FP	θ = 0.2 Power	Mean	SD	SE	Cov	RMSE	TP	FP
								$N_Y = 5e4$								
cisMR-cML-BIC	0.536	0.002	0.125	0.027	0.464	0.125	2.658	0.306	0.940	0.221	0.127	0.029	0.444	0.128	2.412	0.274
cisMR-cML	0.078	0.001	0.095	0.095	0.922	0.095			0.660	0.220	0.090	0.093	0.904	0.092		
GEgger	0.112	0.010	0.121	0.111	0.888	0.121			0.456	0.206	0.121	0.112	0.884	0.121		
GIVW	0.310	0.002	0.126	0.078	0.690	0.126			0.560	0.199	0.126	0.079	0.696	0.126		
LEgger	0.106	0.001	0.074	0.057	0.846	0.074			0.798	0.197	0.075	0.058	0.850	0.075		
MRLDP	0.038	0.000	0.066	NA	NA	0.066			0.666	0.176	0.072	NA	NA	0.076		
MRAID	0.332	-0.003	0.155	NA	NA	0.155			0.798	0.210	0.140	NA	NA	0.140		
MRCUE	0.036	0.002	0.074	0.082	0.964	0.074			0.588	0.183	0.087	0.086	0.926	0.089		
								$N_Y = 1e5$								
cisMR-cML-BIC	0.528	0.000	0.088	0.019	0.472	0.088	2.664	0.306	0.994	0.223	0.089	0.022	0.444	0.091	2.194	0.28
cisMR-cML	0.076	0.001	0.068	0.069	0.924	0.068			0.866	0.221	0.065	0.069	0.904	0.069		
GEgger	0.112	0.007	0.085	0.078	0.888	0.086			0.666	0.203	0.086	0.079	0.880	0.086		
GIVW	0.310	0.001	0.089	0.055	0.690	0.089			0.800	0.198	0.090	0.056	0.708	0.090		
LEgger	0.106	0.000	0.052	0.040	0.846	0.052			0.952	0.196	0.053	0.041	0.848	0.054		
MRLDP	0.038	0.000	0.047	NA	NA	0.047			0.878	0.179	0.056	NA	NA	0.060		
MRAID	0.362	-0.003	0.125	NA	NA	0.125			0.900	0.220	0.117	NA	NA	0.118		
MRCUE	0.036	0.001	0.053	0.061	0.964	0.053			0.902	0.203	0.056	0.062	0.950	0.056		
								$N_Y = 5e5$								
cisMR-cML-BIC	0.516	0.001	0.048	0.009	0.484	0.048	2.688	0.288	0.998	0.215	0.047	0.014	0.378	0.049	1.342	0.322
cisMR-cML	0.072	0.000	0.030	0.035	0.928	0.030			0.998	0.213	0.032	0.036	0.914	0.035		
GEgger	0.112	0.003	0.038	0.035	0.888	0.038			0.998	0.199	0.040	0.037	0.896	0.040		
GIVW	0.310	0.001	0.040	0.025	0.690	0.040			1.000	0.197	0.041	0.026	0.710	0.041		
LEgger	0.106	0.000	0.023	0.018	0.846	0.023			0.998	0.196	0.026	0.021	0.866	0.026		
MRLDP	0.038	0.000	0.021	NA	NA	0.021			0.996	0.194	0.037	NA	NA	0.037		
MRAID	0.438	-0.002	0.063	NA	NA	0.063			0.990	0.210	0.051	NA	NA	0.052		
MRCUE	0.036	0.001	0.024	0.027	0.964	0.024			1.000	0.202	0.029	0.030	0.932	0.029		

*Among 500 simulation replicates, MR.Corr2 often failed to converge thus results are not shown.

Table S13. **Simulation results for weak invalid IV scenario with $r_i = \kappa/\sqrt{N_Y}$, $\kappa = 5$ under the setup of $\rho = 0.6$ in the autoregressive LD structure and 4 invalid IVs, $N_X = 1e4$ samples.** Type-I error (T1E) or power, mean and standard deviation (SD) of estimates, mean standard error (SE) and coverage rate (Cov), root mean squared error (RMSE), mean of true positives (TP) and false positives (FP).

	$\theta = 0$								$\theta = 0.2$							
Method	TIE	Mean	SD	SE	Cov	RMSE	TP	FP	Power	Mean	SD	SE	Cov	RMSE	TP	FP
								$N_Y = 5e4$								
cisMR-cML-BIC	0.042	0.002	0.073	0.033	0.958	0.072	3.988	0.014	0.990	0.200	0.063	0.036	0.956	0.063	3.992	0.016
cisMR-cML	0.004	0.002	0.061	0.068	0.996	0.061			0.840	0.200	0.069	0.072	0.988	0.069		
GEgger	0.120	0.034	0.469	0.430	0.880	0.470			0.154	0.230	0.470	0.431	0.880	0.470		
GIVW	0.318	0.003	0.499	0.305	0.682	0.498			0.340	0.200	0.499	0.306	0.686	0.499		
LEgger	0.110	0.001	0.277	0.211	0.830	0.277			0.170	0.197	0.278	0.211	0.826	0.278		
MRLDP	0.000	0.000	0.068	NA	NA	0.068			0.000	0.050	0.067	NA	NA	0.165		
MRAID	0.118	0.000	0.311	NA	NA	0.310			0.788	0.153	0.317	NA	NA	0.321		
								$N_Y = 1e5$								
cisMR-cML-BIC	0.048	0.004	0.062	0.023	0.952	0.062	3.98	0.02	0.998	0.201	0.073	0.027	0.942	0.073	3.966	0.03
cisMR-cML	0.006	0.002	0.053	0.058	0.994	0.053			0.860	0.203	0.059	0.060	0.988	0.059		
GEgger	0.120	0.024	0.332	0.304	0.880	0.332			0.184	0.220	0.332	0.305	0.880	0.332		
GIVW	0.318	0.002	0.353	0.216	0.682	0.352			0.350	0.199	0.353	0.216	0.688	0.353		
LEgger	0.110	0.001	0.196	0.149	0.830	0.196			0.228	0.196	0.197	0.150	0.820	0.196		
MRLDP	0.000	0.000	0.048	NA	NA	0.048			0.000	0.049	0.046	NA	NA	0.158		
MRAID	0.184	-0.003	0.275	NA	NA	0.274			0.832	0.165	0.266	NA	NA	0.269		
								$N_Y = 5e5$								
cisMR-cML-BIC	0.050	0.003	0.035	0.010	0.950	0.035	3.964	0.03	0.990	0.208	0.073	0.019	0.840	0.073	3.818	0.364
cisMR-cML	0.002	0.001	0.033	0.037	0.998	0.033			0.866	0.210	0.048	0.064	0.980	0.049		
GEgger	0.120	0.011	0.148	0.136	0.880	0.149			0.350	0.206	0.149	0.137	0.882	0.149		
GIVW	0.318	0.001	0.158	0.097	0.682	0.158			0.462	0.198	0.158	0.097	0.688	0.158		
LEgger	0.110	0.000	0.088	0.067	0.830	0.088			0.704	0.196	0.089	0.068	0.820	0.089		
MRLDP	0.000	0.000	0.021	NA	NA	0.021			0.000	0.041	0.019	NA	NA	0.160		
MRAID	0.240	0.001	0.143	NA	NA	0.143			0.856	0.176	0.116	NA	NA	0.118		
MRCUE	0.032	0.001	0.078	0.086	0.968	0.078			0.214	0.099	0.089	0.083	0.750	0.134		

*Among 500 simulation replicates, MR.Corr2 and MR.CUE (when $N_Y = 5e4, 1e5$) often failed to converge thus results are not shown.

Table S14. **Simulation results for weak invalid IV scenario with $r_i = \kappa/\sqrt{N_Y}$, $\kappa = 20$ under the setup of $\rho = 0.6$ in the autoregressive LD structure and 4 invalid IVs, $N_X = 1e4$ samples.** Type-I error (T1E) or power, mean and standard deviation (SD) of estimates, mean standard error (SE) and coverage rate (Cov), root mean squared error (RMSE), mean of true positives (TP) and false positives (FP).

	$\theta = 0$						$\theta = 0.2$					
Method	TIE	Mean	SD	SE	Cov	RMSE	Power	Mean	SD	SE	Cov	RMSE
						$\rho_{XY} = 0$						
cisMR-cML	0.020	0.000	0.023	0.030	0.980	0.023	0.992	0.201	0.024	0.032	0.982	0.024
cisMR-cML-X	0.018	-0.001	0.023	0.028	0.982	0.023	0.996	0.200	0.024	0.031	0.982	0.024
GEgger-X	0.032	-0.001	0.040	0.040	0.968	0.039	0.970	0.198	0.043	0.042	0.956	0.043
GIVW-X	0.036	-0.001	0.020	0.021	0.964	0.020	1.000	0.198	0.021	0.022	0.960	0.021
LEgger-X	0.038	0.002	0.035	0.028	0.846	0.035	0.916	0.199	0.039	0.030	0.828	0.039
						$\rho_{XY} = 0.1$						
cisMR-cML	0.016	0.000	0.023	0.030	0.984	0.023	0.992	0.202	0.024	0.032	0.982	0.024
cisMR-cML-X	0.226	0.008	0.074	0.053	0.774	0.074	0.872	0.210	0.074	0.055	0.766	0.074
GEgger-X	0.042	0.002	0.083	0.073	0.958	0.083	0.784	0.201	0.085	0.074	0.938	0.085
GIVW-X	0.308	0.006	0.080	0.047	0.692	0.081	0.844	0.205	0.080	0.048	0.702	0.080
LEgger-X	0.050	0.002	0.044	0.034	0.858	0.044	0.882	0.199	0.048	0.036	0.854	0.048

Table S15. **Simulation results when SNPs in $\mathcal{I}_X$ and SNPs in $\mathcal{I}_Y$ were either uncorrelated ($\rho_{XY} = 0$) or weakly correlated ($\rho_{XY} = 0.1$).** Type-I error (T1E) or power, mean and standard deviation (SD) of estimates, mean standard error (SE) and coverage rate (Cov), root mean squared error (RMSE).

	$\theta = 0$								$\theta = 0.2$							
Method	TIE	Mean	SD	SE	Cov	RMSE	TP	FP	Power	Mean	SD	SE	Cov	RMSE	TP	FP
$N = 5e5, \kappa = 1$																
cisMR-cML-BIC	0.146	7.31E-04	3.31E-02	9.45E-03	0.854	3.30E-02	3.784	0.064	1.000	2.02E-01	3.49E-02	9.50E-03	0.842	3.49E-02	3.746	0.072
GEgger	0.100	5.81E-03	6.58E-02	6.20E-02	0.900	6.60E-02			0.870	2.06E-01	6.59E-02	6.20E-02	0.896	6.61E-02		
GIVW	0.320	6.28E-04	7.13E-02	4.35E-02	0.680	7.12E-02			0.994	2.01E-01	7.13E-02	4.35E-02	0.686	7.13E-02		
LEgger	0.102	3.60E-04	4.04E-02	3.06E-02	0.828	4.04E-02			0.988	2.00E-01	4.06E-02	3.06E-02	0.824	4.05E-02		
$N = 5e8, \kappa = 1$																
cisMR-cML-BIC	0.038	1.05E-05	3.49E-04	3.30E-04	0.962	3.48E-04	4.000	0.000	1.000	2.00E-01	7.36E-04	3.43E-04	0.952	7.36E-04	3.994	0.046
GEgger	0.110	4.83E-04	6.55E-03	6.16E-03	0.890	6.56E-03			1.000	2.00E-01	6.55E-03	6.16E-03	0.890	6.56E-03		
GIVW	0.328	3.78E-05	7.11E-03	4.32E-03	0.672	7.10E-03			1.000	2.00E-01	7.11E-03	4.32E-03	0.672	7.11E-03		
LEgger	0.114	2.42E-05	3.97E-03	3.00E-03	0.832	3.97E-03			1.000	2.00E-01	3.97E-03	3.00E-03	0.832	3.97E-03		
$N = 5e5, \kappa = 5$																
cisMR-cML-BIC	0.038	4.21E-04	1.12E-02	1.04E-02	0.962	1.12E-02	4.000	0.004	1.000	2.00E-01	1.10E-02	1.06E-02	0.964	1.10E-02	4.000	0.000
GEgger	0.104	2.66E-02	3.26E-01	3.07E-01	0.896	3.27E-01			0.194	2.26E-01	3.26E-01	3.07E-01	0.896	3.27E-01		
GIVW	0.330	1.69E-03	3.55E-01	2.16E-01	0.670	3.55E-01			0.352	2.02E-01	3.55E-01	2.16E-01	0.670	3.55E-01		
LEgger	0.112	1.03E-03	1.98E-01	1.50E-01	0.828	1.98E-01			0.228	2.01E-01	1.98E-01	1.50E-01	0.828	1.98E-01		
$N = 5e8, \kappa = 5$																
cisMR-cML-BIC	0.038	1.05E-05	3.49E-04	3.30E-04	0.962	3.48E-04	4.000	0.000	1.000	2.00E-01	3.50E-04	3.36E-04	0.966	3.49E-04	4.000	0.000
GEgger	0.108	2.33E-03	3.27E-02	3.08E-02	0.892	3.28E-02			1.000	2.02E-01	3.27E-02	3.08E-02	0.892	3.28E-02		
GIVW	0.332	1.43E-04	3.55E-02	2.16E-02	0.668	3.55E-02			1.000	2.00E-01	3.56E-02	2.16E-02	0.666	3.55E-02		
LEgger	0.110	9.67E-05	1.98E-02	1.50E-02	0.830	1.98E-02			1.000	2.00E-01	1.98E-02	1.50E-02	0.830	1.98E-02		

Table S16. **Simulation results with $r_i = \kappa/\sqrt[3]{N}$ under the setup of $\rho = 0.6$ in the autoregressive LD structure and 4 invalid IVs, $N_X = N_Y = N$ samples.** Type-I error (T1E) or power, mean and standard deviation (SD) of estimates, mean standard error (SE) and coverage rate (Cov), root mean squared error (RMSE), mean of true positives (TP) and false positives (FP).

	$\theta = 0$								$\theta = 0.2$							
Method	TIE	Mean	SD	SE	Cov	RMSE	TP	FP	Power	Mean	SD	SE	Cov	RMSE	TP	FP
$N = 5e5, \kappa = 1$																
cisMR-cML-BIC	0.244	4.13E-04	1.04E-02	6.24E-03	0.756	1.04E-02	0.102	0.028	1	2.00E-01	1.06E-02	6.35E-03	0.754	1.06E-02	0.094	0.024
GEgger	0.078	1.20E-03	1.19E-02	1.15E-02	0.922	1.19E-02			1	2.01E-01	1.20E-02	1.17E-02	0.922	1.20E-02		
GIVW	0.146	3.93E-04	1.01E-02	7.82E-03	0.854	1.01E-02			1	2.00E-01	1.02E-02	7.90E-03	0.848	1.02E-02		
LEgger	0.066	2.12E-04	9.38E-03	8.02E-03	0.884	9.37E-03			1	2.00E-01	9.49E-03	8.14E-03	0.878	9.48E-03		
$N = 5e8, \kappa = 1$																
cisMR-cML-BIC	0.242	1.29E-05	3.26E-04	1.95E-04	0.758	3.26E-04	0.024	0.004	1	2.00E-01	3.27E-04	1.99E-04	0.762	3.27E-04	0.014	0.002
GEgger	0.074	3.77E-05	3.73E-04	3.66E-04	0.926	3.75E-04			1	2.00E-01	3.77E-04	3.70E-04	0.918	3.78E-04		
GIVW	0.146	1.24E-05	3.19E-04	2.47E-04	0.854	3.19E-04			1	2.00E-01	3.23E-04	2.50E-04	0.846	3.23E-04		
LEgger	0.070	6.68E-06	2.97E-04	2.54E-04	0.882	2.96E-04			1	2.00E-01	3.00E-04	2.57E-04	0.878	3.00E-04		
$N = 5e5, \kappa = 5$																
cisMR-cML-BIC	0.594	-6.27E-04	3.47E-02	7.66E-03	0.406	3.46E-02	2.374	0.188	0.998	2.00E-01	3.71E-02	7.82E-03	0.398	3.71E-02	2.290	0.214
GEgger	0.094	3.53E-03	3.77E-02	3.55E-02	0.906	3.79E-02			1	2.03E-01	3.78E-02	3.56E-02	0.904	3.79E-02		
GIVW	0.308	5.12E-04	4.03E-02	2.49E-02	0.692	4.03E-02			1	2.00E-01	4.04E-02	2.49E-02	0.698	4.03E-02		
LEgger	0.108	2.87E-04	2.37E-02	1.81E-02	0.850	2.37E-02			1	2.00E-01	2.38E-02	1.81E-02	0.842	2.38E-02		
$N = 5e8, \kappa = 5$																
cisMR-cML-BIC	0.664	2.80E-06	1.15E-03	2.32E-04	0.336	1.15E-03	1.924	0.120	1	2.00E-01	1.28E-03	2.41E-04	0.304	1.28E-03	1.834	0.194
GEgger	0.094	1.03E-04	1.19E-03	1.12E-03	0.906	1.20E-03			1	2.00E-01	1.20E-03	1.13E-03	0.908	1.20E-03		
GIVW	0.308	1.62E-05	1.28E-03	7.87E-04	0.692	1.27E-03			1	2.00E-01	1.28E-03	7.87E-04	0.698	1.28E-03		
LEgger	0.104	9.25E-06	7.49E-04	5.71E-04	0.852	7.49E-04			1	2.00E-01	7.53E-04	5.74E-04	0.846	7.53E-04		

Table S17. **Simulation results with $r_i = \kappa/\sqrt[2]{N}$ under the setup of $\rho = 0.6$ in the autoregressive LD structure and 4 invalid IVs, $N_X = N_Y = N$ samples.** Type-I error (T1E) or power, mean and standard deviation (SD) of estimates, mean standard error (SE) and coverage rate (Cov), root mean squared error (RMSE), mean of true positives (TP) and false positives (FP).

Method	TIE	Mean	SD	SE	Cov	RMSE	TP	FP	Power	Mean	SD	SE	Cov	RMSE	TP	FP
	$\theta = 0$								$\theta = 0.2$							
								$N = 5e5, \kappa = 1$								
cisMR-cML-BIC	0.060	3.90E-04	6.42E-03	6.17E-03	0.940	6.42E-03	0.002	0.002	1	2.00E-01	6.54E-03	6.29E-03	0.940	6.54E-03	0.002	0.002
GEgger	0.038	6.18E-04	9.40E-03	9.92E-03	0.962	9.41E-03			1	2.00E-01	9.45E-03	1.00E-02	0.958	9.45E-03		
GIVW	0.040	3.64E-04	6.26E-03	6.67E-03	0.960	6.26E-03			1	2.00E-01	6.39E-03	6.74E-03	0.952	6.39E-03		
LEgger	0.050	1.94E-04	8.26E-03	7.40E-03	0.900	8.25E-03			1	2.00E-01	8.33E-03	7.53E-03	0.906	8.32E-03		
								$N = 5e8, \kappa = 1$								
cisMR-cML-BIC	0.054	1.15E-05	1.98E-04	1.95E-04	0.946	1.98E-04	0.000	0.000	1	2.00E-01	2.02E-04	1.99E-04	0.942	2.02E-04	0.000	0.000
GEgger	0.040	2.13E-05	2.96E-04	3.14E-04	0.960	2.97E-04			1	2.00E-01	2.99E-04	3.17E-04	0.956	2.99E-04		
GIVW	0.040	1.15E-05	1.98E-04	2.11E-04	0.960	1.98E-04			1	2.00E-01	2.02E-04	2.13E-04	0.952	2.02E-04		
LEgger	0.050	6.03E-06	2.62E-04	2.34E-04	0.902	2.62E-04			1	2.00E-01	2.64E-04	2.38E-04	0.906	2.64E-04		
								$N = 5e5, \kappa = 5$								
cisMR-cML-BIC	0.058	3.90E-04	6.42E-03	6.17E-03	0.942	6.42E-03	0.002	0.002	1	2.00E-01	6.54E-03	6.29E-03	0.940	6.54E-03	0.002	0.002
GEgger	0.038	6.21E-04	9.40E-03	9.92E-03	0.962	9.41E-03			1	2.00E-01	9.45E-03	1.00E-02	0.958	9.45E-03		
GIVW	0.040	3.64E-04	6.26E-03	6.67E-03	0.960	6.26E-03			1	2.00E-01	6.39E-03	6.74E-03	0.952	6.39E-03		
LEgger	0.050	1.94E-04	8.26E-03	7.40E-03	0.900	8.25E-03			1	2.00E-01	8.33E-03	7.53E-03	0.906	8.32E-03		
								$N = 5e8, \kappa = 5$								
cisMR-cML-BIC	0.054	1.15E-05	1.98E-04	1.95E-04	0.946	1.98E-04	0.000	0.000	1	2.00E-01	2.02E-04	1.99E-04	0.942	2.02E-04	0.000	0.000
GEgger	0.040	2.13E-05	2.96E-04	3.14E-04	0.960	2.97E-04			1	2.00E-01	2.99E-04	3.17E-04	0.956	2.99E-04		
GIVW	0.040	1.15E-05	1.98E-04	2.11E-04	0.960	1.98E-04			1	2.00E-01	2.02E-04	2.13E-04	0.952	2.02E-04		
LEgger	0.050	6.03E-06	2.62E-04	2.34E-04	0.902	2.62E-04			1	2.00E-01	2.64E-04	2.38E-04	0.906	2.64E-04		

Table S18. **Simulation results with $r_i = \kappa/N$ under the setup of $\rho = 0.6$ in the autoregressive LD structure and 4 invalid IVs, $N_X = N_Y = N$ samples.** Type-I error (T1E) or power, mean and standard deviation (SD) of estimates, mean standard error (SE) and coverage rate (Cov), root mean squared error (RMSE), mean of true positives (TP) and false positives (FP).

S4 Full results in the second set of simulations

	$\theta = 0$						$\theta = 0.05$					
Method	TIE	Mean	SD	SE	Cov	RMSE	Power	Mean	SD	SE	Cov	RMSE
**Scenario 1 with SNPs in $\mathcal{I}_X$ and $\mathcal{I}_Y$, and corresponding genetic effect sizes specified according to real-data**												
cisMR-cML	0.040	0.002	0.015	0.017	0.960	0.016	0.792	0.050	0.017	0.019	0.962	0.017
cisMR-cML-X	0.076	0.004	0.023	0.019	0.924	0.023	0.820	0.054	0.021	0.019	0.918	0.021
GEgger-X	0.052	-0.001	0.079	0.056	0.948	0.079	0.434	0.053	0.079	0.055	0.910	0.079
GIVW-X	0.134	0.003	0.019	0.015	0.866	0.019	0.880	0.054	0.019	0.015	0.824	0.020
IVW-IND	0.162	0.003	0.021	0.014	0.838	0.022	0.882	0.052	0.022	0.014	0.830	0.022
LEgger	0.034	0.008	0.046	0.040	0.900	0.047	0.224	0.056	0.047	0.039	0.876	0.047
MR.LDP	0.430	4.08E+49	9.03E+50	NA	NA	9.03E+50	0.461	-1.01E+131	2.24E+132	NA	NA	2.24E+132
MRAID	0.042	-0.001	0.021	NA	NA	0.021	0.286	0.017	0.022	NA	NA	0.040
MR.Corr2*	0.348	0.000	0.008	0.003	0.652	0.008	0.380	0.005	0.013	0.003	0.056	0.047
MR.CUE	0.411	-0.001	0.012	0.004	0.589	0.012	0.473	0.004	0.017	0.004	0.054	0.049
**Scenario 2 with SNPs in $\mathcal{I}_X$ and $\mathcal{I}_Y$, and corresponding genetic effect sizes randomly generated**												
cisMR-cML	0.038	2.06E-04	0.029	0.033	0.962	0.029	0.338	0.045	0.027	0.033	0.964	0.028
cisMR-cML-X	0.202	0.005	0.112	0.061	0.798	0.112	0.298	0.044	0.109	0.060	0.792	0.109
GEgger-X	0.234	0.016	1.065	0.667	0.766	1.064	0.226	0.064	1.039	0.653	0.764	1.038
GIVW-X	0.314	0.003	0.161	0.085	0.686	0.161	0.358	0.048	0.157	0.083	0.686	0.157
IVW-IND	0.638	0.009	0.200	0.037	0.362	0.200	0.662	0.054	0.201	0.038	0.362	0.200
LEgger	0.098	-0.014	0.298	0.227	0.858	0.298	0.102	0.032	0.294	0.221	0.854	0.295
MR.LDP	0.128	-3.37E+98	7.55E+99	NA	NA	7.55E+99	0.130	-3.32E+98	7.44E+99	NA	NA	7.44E+99
MRAID	0.126	-0.002	0.057	NA	NA	0.057	0.148	0.007	0.061	NA	NA	0.075
MR.Corr2**	0.070	1.44E-04	0.003	0.001	0.930	0.003	0.150	4.53E-04	0.004	0.001	0.006	0.050
MR.CUE***	0.161	-0.001	0.032	0.013	0.839	0.032	0.151	1.90E-04	0.027	0.014	0.158	0.057

*Among 500 simulation replicates, the traceplots for MR.Corr2 gibbs sampler were flat at zero in 69, 142 replicates when $\theta = 0$ and 0.05 respectively.
**Among 500 simulation replicates, the traceplots for MR.Corr2 gibbs sampler were flat at zero in 457, 460 replicates when $\theta = 0$ and 0.05 respectively.
***Among 500 simulation replicates, the traceplots for MR.CUE gibbs sampler were flat at zero in 122, 116 replicates when $\theta = 0$ and 0.05 respectively.

Table S19. **Simulation results for the second set of simulations based on real LD patterns involving 50 randomly selected proteins from the proteome-wide application.** Type-I error (T1E) or power, mean and standard deviation (SD) of estimates, mean standard error (SE) and coverage rate (Cov), root mean squared error (RMSE).

S5 Sensitivity analysis in the second data application using different p-value thresholds

In the main proteome-wide analysis discussed in the main text, we proposed to use 5×10^{-6} as the COJO p-value (of the conditional/joint effect) threshold to select SNPs in $\mathcal{I}_X$ and SNPs in $\mathcal{I}_Y$. In this section, we tried three other thresholds: 5×10^{-8}, 5×10^{-4} and 5×10^{-3}, which had 605, 979 and 963 proteins remained in the subsequent analysis respectively.

As in the main text, we used the Benjamini-Hochberg approach to account for multiple testing and reported significant MR findings with a false discovery rate (FDR) less than

0.05. First, using a stringent threshold of 5×10^{-8}, cisMR-cML identified 6 including the same three proteins: PCSK9, COLEC11, FGFR1, and three new ones: ERAP2, AGT, HGFAC, where ERAP2 had colocalization evidence. The inflation factor was slightly inflated around 1.08. This could be partially due to the reason that we omitted some SNPs in $\mathcal{I}_Y$ in the analysis, which will lead to inflated type-I error as shown in our simulation studies. Second, using a relatively loose threshold, cisMR-cML only identified FGFR1 when using 5×10^{-4}, and only identified APOE when using 5×10^{-3}. The inflation factors were deflated around 0.71 and 0.45 respectively. This could be partially due to the inclusion of irrelevant SNPs in the analysis, which resulted in a reduced power as illustrated in Table S9. The Q-Q plot for cisMR-cML using different thresholds to select SNPs in COJO is shown in Fig. S1.

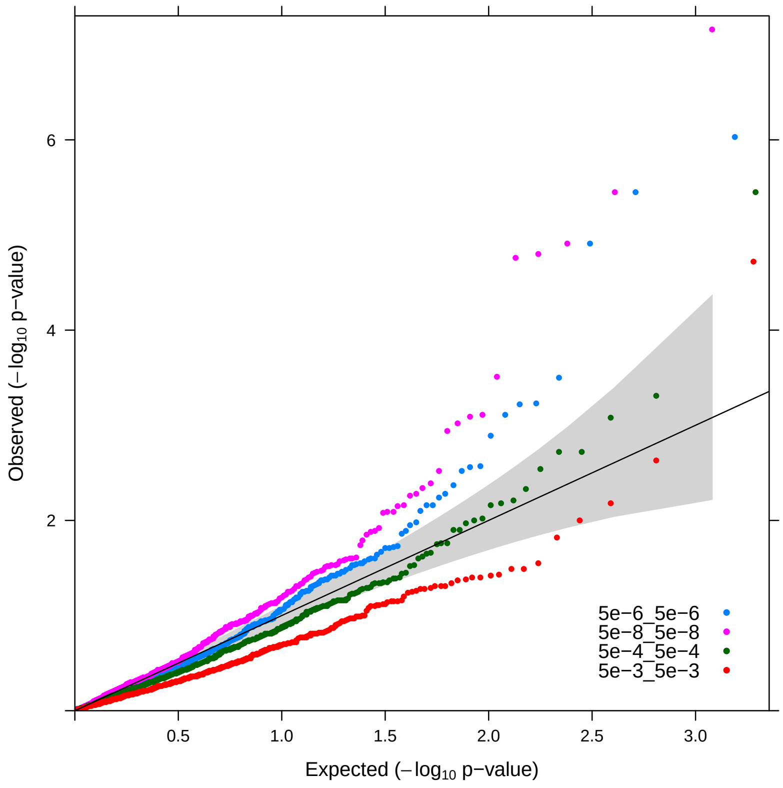

Figure S1. Q-Q plots of $-\log_{10}$ p-value for cisMR-cML (with data perturbation) under different p-value thresholds to select SNPs in COJO. The p-values are calculated based on two-sided cisMR-cML data perturbation test without multiple testing adjustment.

We also show the Q-Q plots for other methods and their corresponding inflation factors in Fig. S2. It is clear that ignoring the uncertainty in model selection in cisMR-cML-BIC

yielded highly inflated Q-Q plots, compared to the one with data perturbation (referred to as cisMR-cML in the plots). Alternative methods that modeled the marginal effect estimates of SNPs in $\mathcal{I}_X$, including GIVW-X, GEgger-X, and Wald-ratio test, all yielded inflation, while LEgger had deflation and no significant protein was identified by LEgger regardless of the p-value threshold we used.

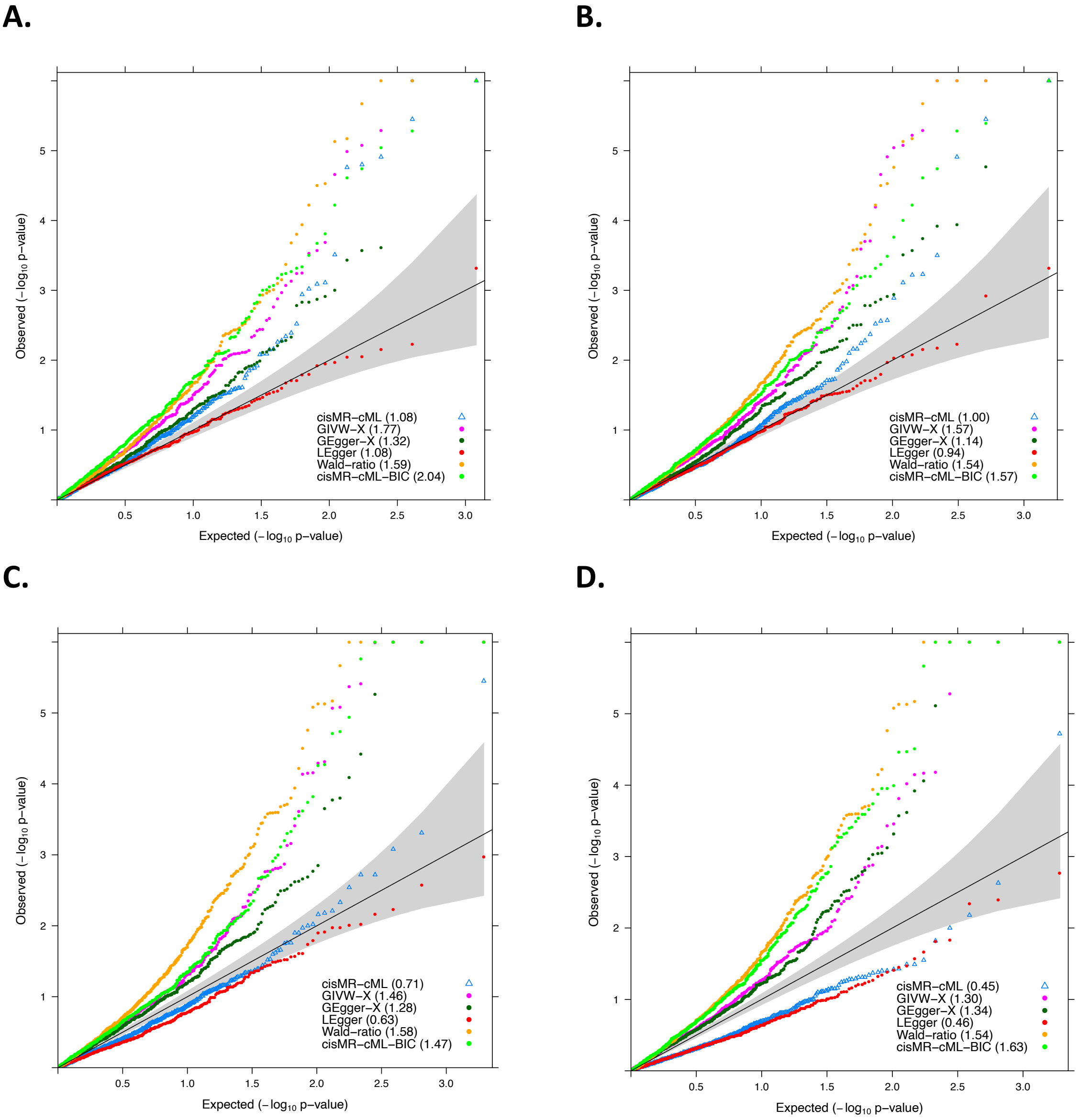

Figure S2. Q-Q plots of $-\log_{10}$ p-value for different methods under different p-value thresholds to select SNPs in COJO. **Panel A:** 5×10^{-8}; **Panel B:** 5×10^{-6}; **Panel C:** 5×10^{-4}; **Panel D:** 5×10^{-3}. Numbers in parentheses are inflation factors. The p-values are calculated based on corresponding two-sided MR tests without multiple testing adjustment.

References

[1] Xue, H., Shen, X. & Pan, W. Constrained maximum likelihood-based Mendelian randomization robust to both correlated and uncorrelated pleiotropic effects. *The American Journal of Human Genetics* **108**, 1251–1269 (2021).

[2] Guo, Z., Kang, H., Tony Cai, T. & Small, D. S. Confidence intervals for causal effects with invalid instruments by using two-stage hard thresholding with voting. *Journal of the Royal Statistical Society Series B: Statistical Methodology* **80**, 793–815 (2018).

[3] Rees, J. M., Wood, A. M., Dudbridge, F. & Burgess, S. Robust methods in Mendelian randomization via penalization of heterogeneous causal estimates. *PloS one* **14**, e0222362 (2019).
