# Supplementary material for: A robust cis-Mendelian randomization method with application to drug target discovery
Source: Nat Commun. 2024 Jul 18;15:6072. doi: 10.1038/s41467-024-50385-y (PMC11258283; doi:10.1038/s41467-024-50385-y)
Supplement: Supplementary file 3 — Reporting Summary [file 41467_2024_50385_MOESM3_ESM.pdf]

nature portfolio

Corresponding author(s): Zhaotong Lin

Last updated by author(s): Jun 24, 2024

Reporting Summary

Nature Portfolio wishes to improve the reproducibility of the work that we publish. This form provides structure for consistency and transparency in reporting. For further information on Nature Portfolio policies, see our Editorial Policies and the Editorial Policy Checklist.

Statistics

For all statistical analyses, confirm that the following items are present in the figure legend, table legend, main text, or Methods section.

n/a	Confirmed	
☐	☒	The exact sample size (*n*) for each experimental group/condition, given as a discrete number and unit of measurement
☒	☐	A statement on whether measurements were taken from distinct samples or whether the same sample was measured repeatedly
☐	☒	The statistical test(s) used AND whether they are one- or two-sided<br>*Only common tests should be described solely by name; describe more complex techniques in the Methods section.*
☒	☐	A description of all covariates tested
☐	☒	A description of any assumptions or corrections, such as tests of normality and adjustment for multiple comparisons
☐	☒	A full description of the statistical parameters including central tendency (e.g. means) or other basic estimates (e.g. regression coefficient) AND variation (e.g. standard deviation) or associated estimates of uncertainty (e.g. confidence intervals)
☐	☒	For null hypothesis testing, the test statistic (e.g. *F*, *t*, *r*) with confidence intervals, effect sizes, degrees of freedom and *P* value noted<br>*Give P values as exact values whenever suitable.*
☒	☐	For Bayesian analysis, information on the choice of priors and Markov chain Monte Carlo settings
☒	☐	For hierarchical and complex designs, identification of the appropriate level for tests and full reporting of outcomes
☐	☒	Estimates of effect sizes (e.g. Cohen's *d*, Pearson's *r*), indicating how they were calculated

Our web collection on statistics for biologists contains articles on many of the points above.

Software and code

Policy information about availability of computer code

Data collection	No software was used.
Data analysis	Software used for analysis includes R v3.6.0, R v.4.2.2, PLINK2 (https://www.cog-genomics.org/plink/2.0/);<br>GCTA-COJO (https://yanglab.westlake.edu.cn/software/gcta/#COJO) (version 1.92.3beta3)<br>The following R packages were used:<br>cisMR-cML is available on https://github.com/ZhaotongL/cisMRcML (version 1.0);<br>LDA-Egger is available on https://rbarfield.github.io/Barfield_website/pages/Rcode.html;<br>GEgger, GIVW, IVW-IND, Egger-IND and cML-IND are available in R package MendelianRandomization (version 0.9.0);<br>MR.LDP is available on https://github.com/QingCheng0218/MR.LDP;<br>MR.Corr2 is available on https://github.com/QingCheng0218/MR.Corr2;<br>MR.CUE is available on https://github.com/QingCheng0218/MR.CUE (version 1.0);<br>MRAID is available on https://github.com/yuanzhongshang/MRAID;<br>Colocalization was implemented in R pacakge coloc (version 5.2.3).

For manuscripts utilizing custom algorithms or software that are central to the research but not yet described in published literature, software must be made available to editors and reviewers. We strongly encourage code deposition in a community repository (e.g. GitHub). See the Nature Portfolio guidelines for submitting code & software for further information.

Data

Policy information about availability of data

All manuscripts must include a data availability statement. This statement should provide the following information, where applicable:

- Accession codes, unique identifiers, or web links for publicly available datasets
- A description of any restrictions on data availability
- For clinical datasets or third party data, please ensure that the statement adheres to our policy

The GWAS summary datasets used in the real data analysis are all publicly available at the URLs below.
ARIC pQTL, http://nilanjanchatterjeelab.org/pwas/;
GWAS Catalog studies GCST003116 and GCST005194 for coronary artery disease, https://www.ebi.ac.uk/gwas/home
Neale lab UK Biobank round 2 GWAS of LDL and testosterone, https://www.nealelab.is/uk-biobank/.
The UK Biobank individual-level data are available under restricted access. Researchers can apply for access at https://www.ukbiobank.ac.uk/. Access to UK Biobank individual-level data in this study was approved through UKB Application 35107.
The processed pQTL data used in the real data application are available at https://doi.org/10.6084/m9.figshare.25411957

Research involving human participants, their data, or biological material

Policy information about studies with human participants or human data. See also policy information about sex, gender (identity/presentation), and sexual orientation and race, ethnicity and racism.

Reporting on sex and gender	No sex- or gender-based analyses were conducted
Reporting on race, ethnicity, or other socially relevant groupings	GWAS summary statistics and LD reference panel were mainly based on European.
Population characteristics	We identified populations using self reported and genetic ancestry estimates provided by UKB
Recruitment	N/A (For UK Biobank, please see: https://www.ukbiobank.ac.uk/enable-your-research)
Ethics oversight	N/A (For UK Biobank, please see: https://www.ukbiobank.ac.uk/learn-more-about-uk-biobank/about-us/ethics)

Note that full information on the approval of the study protocol must also be provided in the manuscript.

Field-specific reporting

Please select the one below that is the best fit for your research. If you are not sure, read the appropriate sections before making your selection.

☒ Life sciences ☐ Behavioural & social sciences ☐ Ecological, evolutionary & environmental sciences

For a reference copy of the document with all sections, see nature.com/documents/nr-reporting-summary-flat.pdf

Life sciences study design

All studies must disclose on these points even when the disclosure is negative.

Sample size	We used publicly available GWAS summary data that had pre-determined sample sizes. For the LD reference panel, 337426 individuals were used.
Data exclusions	We didn't exclude individual from GWAS summary data. For the LD reference panel, we excluded related and non White-British ancestry individuals. This exclusion criteria was pre-established, and is commonly done to ensure the LD reference panel has a similar ancestry background as the GWAS data.
Replication	The final software and replication codes are made available through Github repositories https://github.com/ZhaotongL/cisMRcML and https://github.com/ZhaotongL/cisMR-paper.
Randomization	Randomization was not applicable to the study as this study was not designed to study treatment effects.
Blinding	Blinding was not applicable to the study as this study was not designed to study treatment effects

Reporting for specific materials, systems and methods

We require information from authors about some types of materials, experimental systems and methods used in many studies. Here, indicate whether each material, system or method listed is relevant to your study. If you are not sure if a list item applies to your research, read the appropriate section before selecting a response.

Materials & experimental systems

n/a	Involved in the study
☒	☐ Antibodies
☒	☐ Eukaryotic cell lines
☒	☐ Palaeontology and archaeology
☒	☐ Animals and other organisms
☒	☐ Clinical data
☒	☐ Dual use research of concern
☒	☐ Plants

Methods

n/a	Involved in the study
☒	☐ ChIP-seq
☒	☐ Flow cytometry
☒	☐ MRI-based neuroimaging

Plants

Seed stocks	*Report on the source of all seed stocks or other plant material used. If applicable, state the seed stock centre and catalogue number. If plant specimens were collected from the field, describe the collection location, date and sampling procedures.*
Novel plant genotypes	*Describe the methods by which all novel plant genotypes were produced. This includes those generated by transgenic approaches, gene editing, chemical/radiation-based mutagenesis and hybridization. For transgenic lines, describe the transformation method, the number of independent lines analyzed and the generation upon which experiments were performed. For gene-edited lines, describe the editor used, the endogenous sequence targeted for editing, the targeting guide RNA sequence (if applicable) and how the editor was applied.*
Authentication	*Describe any authentication procedures for each seed stock used or novel genotype generated. Describe any experiments used to assess the effect of a mutation and, where applicable, how potential secondary effects (e.g. second site T-DNA insertions, mosiacism, off-target gene editing) were examined.*
